# Iron Biogeochemistry in the High Latitude North Atlantic Ocean

**DOI:** 10.1038/s41598-018-19472-1

**Published:** 2018-01-19

**Authors:** Eric P. Achterberg, Sebastian Steigenberger, Chris M. Marsay, Frédéric A. C. LeMoigne, Stuart C. Painter, Alex R. Baker, Douglas P. Connelly, C. Mark Moore, Alessandro Tagliabue, Toste Tanhua

**Affiliations:** 1 Earth and Ocean Science, National Oceanography Centre Southampton, University of Southampton, Southampton, SO14 3ZH UK; 2 GEOMAR Helmholtz Centre for Ocean Research Kiel, Kiel, 24148 Germany; 3 National Oceanography Centre, Southampton, SO14 3ZH UK; 4 School of Environmental Science, University of East Anglia, Norwich, NR4 7TJ UK; 5 Skidaway Institute of Oceanography, University of Georgia, Savannah, GA 31411 USA; 6 School of Environmental Sciences, University of Liverpool, Liverpool, L69 3GB UK

## Abstract

Iron (Fe) is an essential micronutrient for marine microbial organisms, and low supply controls productivity in large parts of the world’s ocean. The high latitude North Atlantic is seasonally Fe limited, but Fe distributions and source strengths are poorly constrained. Surface ocean dissolved Fe (DFe) concentrations were low in the study region (<0.1 nM) in summer 2010, with significant perturbations during spring 2010 in the Iceland Basin as a result of an eruption of the Eyjafjallajökull volcano (up to 2.5 nM DFe near Iceland) with biogeochemical consequences. Deep water concentrations in the vicinity of the Reykjanes Ridge system were influenced by pronounced sediment resuspension, with indications for additional inputs by hydrothermal vents, with subsequent lateral transport of Fe and manganese plumes of up to 250–300 km. Particulate Fe formed the dominant pool, as evidenced by 4–17 fold higher total dissolvable Fe compared with DFe concentrations, and a dynamic exchange between the fractions appeared to buffer deep water DFe. Here we show that Fe supply associated with deep winter mixing (up to 103 nmol m^−2^ d^−1^) was at least ca. 4–10 times higher than atmospheric deposition, diffusive fluxes at the base of the summer mixed layer, and horizontal surface ocean fluxes.

## Introduction

Primary productivity, species composition and trophic structure of planktonic communities are controlled in many ocean regions by the availability of light and macronutrients, i.e. nitrogen (N), phosphorus (P) and silicon (Si)^1–3^. However, there is now overwhelming evidence that the availability of the micronutrient iron (Fe) plays a critical role in regulating phytoplankton primary productivity and microbial diversity in the major High Nitrate Low Chlorophyll (HNLC) regions of the sub-Arctic and equatorial Pacific^4,5^ and Southern Ocean^6,7^. These regions account for 40% of the world’s ocean and are replete with macronutrients but have low productivity as a result of a limited supply of Fe, intensified by a low solubility of the thermodynamically favoured Fe(III) redox state^8^. The important role of Fe for microbial organisms is linked to its obligatory requirement in enzymes involved in photosynthesis, respiration, nitrate reduction and nitrogen fixation^9–11^.

Iron and macronutrients are supplied to surface oceans via the atmospheric transport and deposition of dust and volcanic ash^12–14^, lateral transport from continental shelf regions^15–17^, as well as by upwelling, entrainment or mixing of deeper waters relatively enriched through remineralisation of sinking particles^18,19^. Hydrothermal vents are now also recognised as important sources of Fe to the deep ocean, and also contributing to surface ocean productivity^20,21^. These sources supply new nutrients and Fe to the surface mixed layer. The relative importance of atmospheric, lateral and deep ocean sources of nutrients and trace metals to the surface mixed layer will vary both spatially and temporally, with different model studies providing different emphases on the balance of the fluxes^22–24^. In the oceanic surface mixed layer, macronutrients and trace elements are recycled from living matter to sustain regenerated biological production^25,26^.

Despite some early indications of the contrary^27^, Fe supply to the North Atlantic through enhanced deposition of atmospheric dust originating in the Saharan and Sahel deserts^12^ has typically been assumed to be sufficient to meet microbial demands^18^. However, a decrease in surface water dissolved Fe (DFe) concentrations with increasing latitude has been reported for the North Atlantic^28^, as a consequence of decreasing atmospheric Fe inputs^23^. According to deposition models, the dust derived supply of Fe to the high latitude North Atlantic is very low^12^, and comparable to the HNLC North Pacific, although there is evidence for influence from regional dust storms^29^. Recent work has subsequently provided clear indications of Fe limitation in the sub-polar North Atlantic towards the peak of the spring bloom and during post-bloom stages^30–32^. Iron addition bioassay experiments have demonstrated Fe stress of the resident phytoplankton community in summer in both the Iceland Basin (IB) and Irminger Basin (IRB)^30,32^. Following a pronounced annual spring bloom, summer time conditions in the high latitude North Atlantic thus appear comparable to those in the classical HNLC regions, with Fe limitation in this region appearing to be seasonal.

There is a paucity of Fe data for the high latitude North Atlantic, and in particular deep ocean Fe profiles and speciation data are lacking, hampering accurate Fe simulations using biogeochemical models. Previous work has shown that DFe displays a nutrient-type profile in the high latitude North Atlantic with upper water column DFe concentrations of 0.07–0.1 nM and increasing to 0.6–0.8 nM at depths below 900 m^24,27,33^. Furthermore, surface water concentrations of Fe binding ligands (L) in excess of DFe (L′ = L-DFe) ranging between 0.37 and 0.94 nM have been observed during cruises in the IB in 2007 and 2009^34^.

In recent years, the high latitude North Atlantic Ocean has become a focus for research into the role of Fe in ocean productivity and export^25,26,30,32^. This ocean region is a major component of the oceanic carbon cycle and features sites of deep-water formation (North Atlantic Deep Water;^35,36^). Therefore the high latitude North Atlantic has a particular importance for atmospheric CO_2_ sequestration, as any residual nitrate entrained into the formation of deep water represents a lowering of the efficiency of the biological carbon pump^37^. Quantification of the various sources of Fe to the surface ocean is required to establish the relative importance of the different Fe supplies to the microbial communities, which then allows projections to be made of future productivity under different climate scenarios with changed Fe supplies from the various sources. This study is part of the Irminger Basin Iron Study (IBIS) and presents the distribution of dissolved and total dissolvable Fe and the quantification of their various sources to the surface ocean of the high latitude North Atlantic.

## Methods

**Figure 1:**
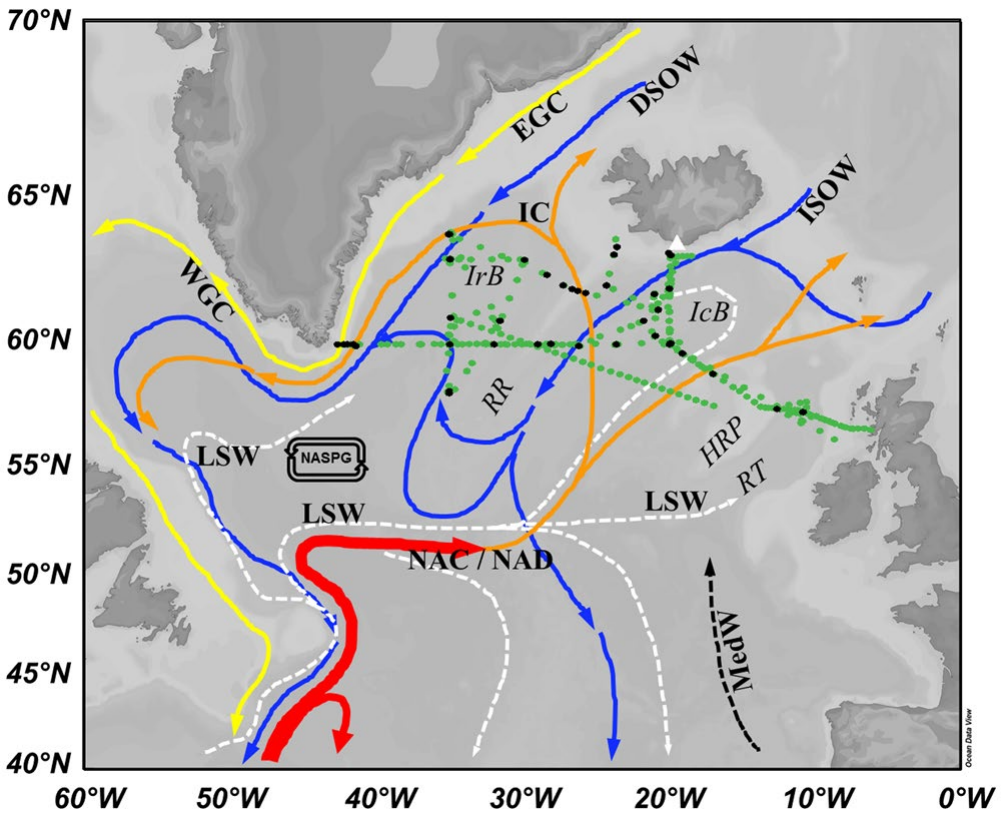
Schematic of the circulation in the North Atlantic, reproduced and modified from the IFM-GEOMAR SFB460 overlaid with the sampling stations of D350, 351 and 354 (magenta: CTD, cyan: underway) and the position of the Eyjafjallajökull volcano (white triangle). Red and yellow: Near-surface currents. Dashed black and white: Intermediate currents. Blue: Near-bottom currents. Abbreviations are: NAC/NAD: North Atlantic Current/Drift, LSW: Labrador Sea Water, ISOW: Iceland Scotland Overflow Water, DSOW: Denmark Strait Overflow Water, NASPG: North Atlantic Sub-Polar Gyre, RT: Rockall Trough, HRP: Hatton-Rockall Plateau, IC: Irminger Current, RR: Reykjanes Ridge, IRB: Irminger Basin and IB: Iceland Basin. Map was produced using Ocean Data View (Schlitzer, R., Ocean Data View, odv.awi.de, 2017).

Water samples were collected from 41 full depth CTD casts and 195 underway surface stations in the high latitude North Atlantic Ocean during cruises D350, D351 and D354 on board RRS *Discovery* as part of the IBIS programme. The cruises took place in spring and summer 2010 (Spring: April 26-May 9 (D350) and May 10–28 (D351); Summer: July 4-August 11 (D354)). The samples were collected in the shelf regions off Iceland (D350, D351, D354) and Greenland (D354), and in the IB (D350, D351, D354) and IRB (D350, D354) (Fig. 1). The spring cruises D350 and D351 were undertaken serendipitously during the explosive eruption phase of the Eyjafjallajökull volcano, and the summer cruise sailed following the eruption. All data is available from the British Oceanographic Data Centre.

Full details of all materials and methods are provided in the supplementary information (SI), and here we provide a brief outline. Surface seawater was pumped from a depth of ca. 3 m using a trace metal clean towed fish and a Teflon bellows pump, and depth profiles were obtained using a Ti CTD rosette frame equipped with trace metal clean water samplers. Samples were collected filtered (0.2 µm) and unfiltered, and acidified to pH 1.9. The storage of the acidified unfiltered samples with subsequent analysis after 18 months yielded total dissolvable Fe (TDFe) and manganese (TDMn) concentrations, which include dissolved (0.2 µm filtered) and an acid leachable fraction of the particulate pool. Manganese was used as a tracer for benthic or seafloor Fe inputs^38,39^.

Dissolved Fe and manganese (DMn) were analysed using ICP-MS with isotope dilution for quantification of Fe and standard addition for Mn^40^. The accuracy and precision of the method was assessed by analyses of SAFe (Sampling and Analysis of Fe) reference samples (http://www.geotraces.org/science/intercalibration/322-standards-and-reference-materials). The values determined using the ICP-MS method showed good consistency with the reported consensus values for DFe and DMn (Table S1). Nutrients were analysed using an auto-analyser following standard procedures^41^.

During the spring, aerosol samples were collected using a high volume aerosol collector (Tisch TSP), and in summer separation of particles into aerodynamic diameters greater than or less than 1 μm was achieved using a Sierra-type cascade impactor^13^. Soluble Fe was obtained following a ammonium acetate leach of the aerosol filters and analysed by ICP-OES^13^. Here we report on the dry deposition fluxes of soluble Fe (F_dry_, nmol m^−2^ d^−1^) as the product of their aerosol concentrations (C_aero_, pmol m^−3^) and a dry deposition velocity (v_d_).

$${{\rm{F}}}_{{\rm{dry}}}={{\rm{C}}}_{{\rm{aero}}}{{\rm{v}}}_{{\rm{d}}}$$

Values of v_d_ were set to 1 and 0.1 cm s^−1^ for the coarse (>1 μm) and fine (<1 μm) aerosol modes, respectively (summer cruise), and 0.7 cm s^−1^ for the spring cruises (see SI for details on choice of v_d_).

Turbulent diffusivity (K) was calculated from turbulent kinetic energy dissipation using data obtained using a free-fall microstructure shear profiler; full details of the approach are provided in Painter, *et al*.^42^. Vertical diffusive fluxes of DFe were subsequently estimated from the turbulent diffusivity and profiles of DFe.

Horizontal surface ocean Fe fluxes were calculated for both the IB and IRB following previous work^43–45^. The Fe fluxes have been estimated from the gradients of DFe and TDFe observed along transects from shelf regions to the open ocean and over the Reykjanes Ridge (transects A, B, C; Fig. S1) to the waters of the IB and IRB (Figs S2 and S3), using reported estimates of horizontal diffusivity. In the high latitude North Atlantic, diffusivities were determined from drifters and the Parallel Ocean Program model that includes eddy-induced mixing, and varied from 0 to 3 × 10^7^ cm^2^ s^−1^ in both North/South and West/East components^46^.

Winter convective fluxes were obtained from examinations of individual Argo float data from the IRB and IB in winter 2010, which allowed determination of the mixed layer depths using criteria by de Boyer Montégut, *et al*.^47^. The obtained winter mixed layer depths were 200 m for the IRB and 350 m for the IB. Dissolved Fe concentrations from profiles were then integrated to the depth of winter mixing to provide an estimate of convective inputs as previously^30^.

Observations are compared against state of the art modelling using the global ocean biogeochemical model PISCES^48^. This model is well suited to examining the results obtained in this study as it represents one of the better performing global Fe models^20^ and includes a wide range of Fe sources (dust, sediment, hydrothermal and river) alongside complex representations of scavenging and ligand dynamics^49^. Here we use a range of simulations with varying hydrothermal Fe inputs as described in Tagliabue and Resing^50^. In short, these test two different types of scenarios, one where the gross hydrothermal input flux of Fe is varied and a second where there is a flux of Fe stabilising ligands from the hydrothermal system.

## Results and Discussion

### Hydrography in study region

The sampling was conducted in the high latitude North Atlantic Ocean, including the IB and IRB, the Reykjanes Ridge and Rockall Trough regions and the Hatton-Rockall Plateau (Fig. 1). The North Atlantic sub-polar gyre flows through the IB and IRB, and has a cyclonic circulation. The North Atlantic Drift (NAD) forms part of the southern limb of the gyre and flows northward into the central part of our study region (between about 10°W and 30°W) as the main near surface current reaching depths up to 1000 m in the IB^51^. The NAD flows towards the Faroe Islands and then feeds the Norwegian current to the north past Iceland^52^. The broad NAD forms an extension of the North Atlantic Current (NAC), which also feeds the Irminger Current, and itself is fed by the Gulf Stream. The salinities of the NAD range between 35.2 and 35.7 and its water temperatures are typically warmer than the surrounding waters^53^. In the eastern region of the IRB, the circulation is dominated by the Irminger Current which deflects west in the northern parts of the IRB and then heads southwards as part of the cyclonic subpolar gyre circulation. The East Greenland Current (EGC) forms a cold and fresh southward flowing western boundary current in the western IRB, with reported temperatures in late summer in the surface layer ranging between 3.5 and 5.5 °C and salinities between 30 and 34^54^.

Characteristics of the water masses in the study region^55^ in addition to NAD, include Labrador Sea Water (LSW; θ 3–3.6 °C; S ~34.86) which is present at depths ranging from subsurface to 2000 m in the IRB and 800 to 2000 m in the IB. The Iceland-Scotland Overflow Water (ISOW; θ 2–3 °C; S 34.9–35.1) flows southwards at depths below 1500 m into the IB from the Arctic. The ISOW joins the cold Denmark Strait Overflow Water (DSOW; θ 0–1.4 °C, S ~34.8) which is prominent in the IRB as a deep water mass below 2000 m, both forming part of the North Atlantic Deep water (NADW; θ 2.1–3.5 °C, S 34.9–35.0). The NADW is found in the IB and IRB at depths below 1000 m^55^.

### Surface water DFe, TDFe concentrations

**Figure 2:**
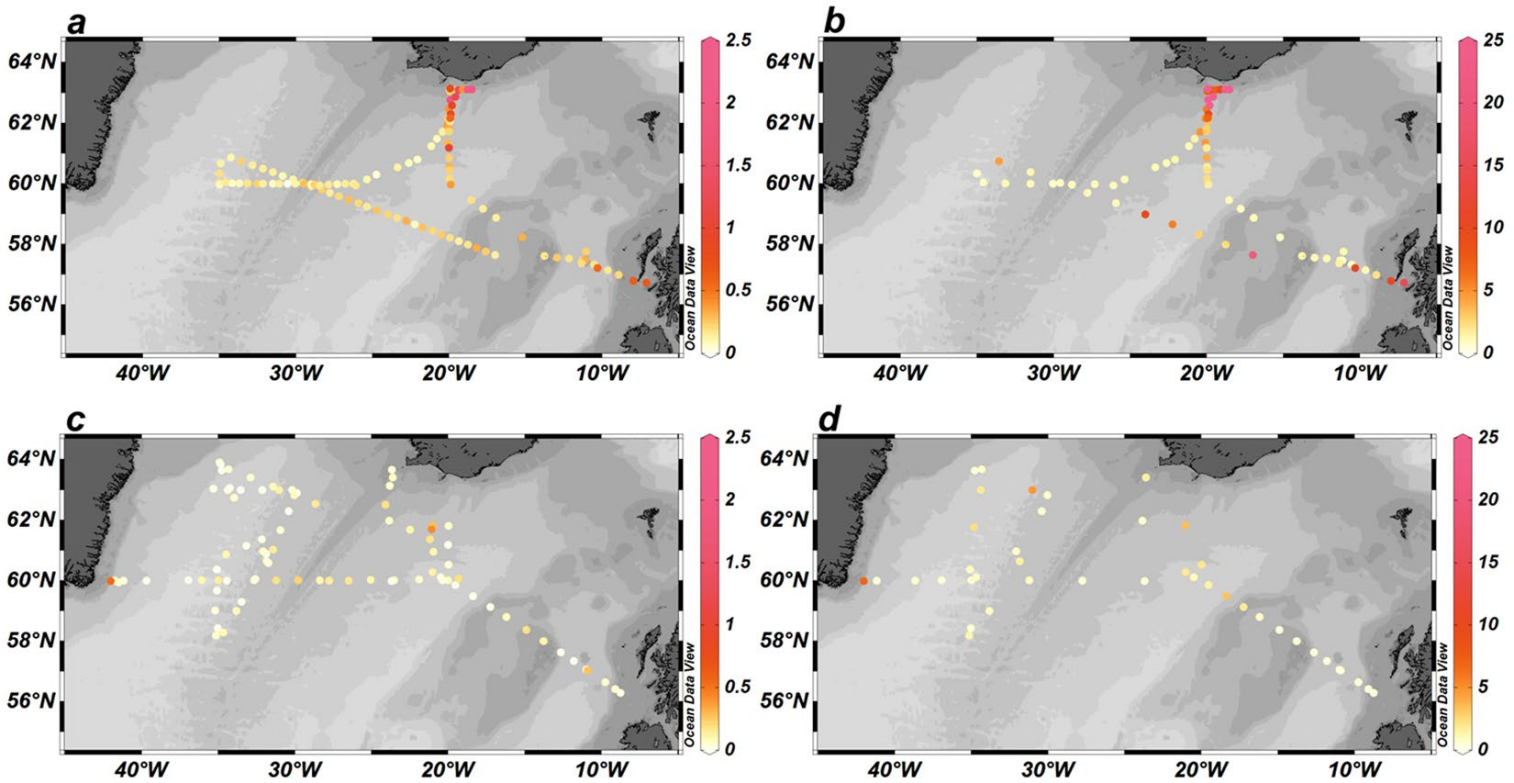
Surface water concentrations in nM of DFe (**a**) and TDFe (**b**) during spring 2010 and of DFe (**c**) and TDFe (**d**) during summer 2010. On Fig. 2b, TDFe reached 218 nM on the Icelandic shelf right under the volcanic plume (not indicated by colour bar). Figures were produced using Ocean Data View (Schlitzer, R., Ocean Data View, odv.awi.de, 2017).

On the cruises we sampled a total of 195 surface stations in the IB and IRB using the towed fish. The ranges and average surface water concentrations at off shore stations (water depths >400 m) for the whole region and the separate IB and IRB are presented in Table 1. Surface water DFe concentrations within the high latitude North Atlantic were modest in spring, with an average DFe of 0.209 ± 0.172 nM. Somewhat enhanced concentrations were observed in the IB (0.243 ± 0.190 nM) as a result of ash-derived Fe inputs from the Eyjafjallajökull volcanic eruption^56^, which caused very high DFe values of 2.5 nM (and up to 218 nM TDFe) directly under the plume (63.1°N, 18.5°W) in the immediate vicinity of the Icelandic coast (Fig. 2a,b), in contrast to more typical concentrations in Icelandic coastal surface waters of ca. 0.2–0.6 nM DFe and 0.5–1.5 nM TDFe (63°N, 19–23°W; cruise D354, Fig. 2c,d). High DFe concentrations (>0.5 nM) were also found on the Scottish Shelf (Fig. 2a), likely due to benthic inputs as a result of reductive dissolution of sedimentary Fe oxy-hydroxides^57^. Enhanced dissolved DMn concentrations (8.73 nM) were also observed on this shelf, with Mn acting as a tracer for benthic inputs^38^. Average DFe concentrations in the IRB in spring (0.124 ± 0.056 nM) were typical of reported surface water concentrations (ca. 0.09 nM) for the high latitude North Atlantic^30,33^. The surface water concentrations of DFe showed a pronounced spatial variability in the open ocean areas (Fig. 2a), which may be related to the spatial and temporal variability in ash deposition^56^ and possibly mesoscale and (sub-) mesoscale processes, including fronts and eddies^58^. Similar patterns were observed for the TDFe distributions (Fig. 2b). However, in the open ocean regions there was no significant relationship between the DFe and TDFe distributions (bivariate Pearson’s Correlation, 0.05 significance level).

**Table 1:** Ranges and average concentration values for DFe, TDFe and nitrate in surface waters of the high latitude North Atlantic (excluding shelf waters).

	Range	Average all (±SD)	Average IRB (±SD)	Average IB (±SD)	t-test (IRB vs IB)	t-test (seasonal comparison IB)	t-test (seasonal comparison IRB)
**Spring cruise D350**							
DFe (nM)	BD – 0.98	0.209(±0.172) n = 74	0.124(±0.056) n = 21	0.243(±0.190) n = 53	P = 0.000	P = 0.000	P = 0.156
TDFe (nM)	0.62–23.26	3.871(±6.166) n = 42	1.454(±1.330) n = 7	4.354(±6.640) n = 35	P = 0.012	P = 0.012	P = 0.443
NO_3_ (µM)	4.94–14.45	10.02(±2.39) n = 71	12.66(±0.97) n = 20	8.99(±1.94) n = 51	P = 0.000	P = 0.000	P = 0.000
**Summer cruise D354**							
DFe (nM)	BD – 0.53	0.091(±0.197) n = 73	0.087(±0.234) n = 48	0.099(±0.095) n = 25	P = 0.377		
TDFe (nM)	0.358–6.81	1.450(±1.424) n = 30	1.364(±1.599) n = 20	1.623(±1.043) n = 10	P = 0.299		
NO_3_ (µM)	BD – 6.47	2.25(±1.74) n = 61	2.97(±1.57) n = 42	0.65(±0.76) n = 19	P = 0.000		

A regional concentration comparison between the Iceland (IB) and Irminger (IRB) Basins is presented, as well as a seasonal comparison between the spring and summer cruises of 2010; Student t-test was undertaken with a 95% significance threshold. BD: below detection limit.

During the summer cruise (Fig. 2c), open ocean DFe concentrations for the whole region were lower with an average of 0.091 ± 0.197 nM. Here, the IB (0.099 ± 0.095 nM) and coastal stations off Scotland and Iceland (Fig. 2c) did not show enhanced DFe concentrations as in spring, with the decrease in concentrations presumably due to lower atmospheric inputs following the cessation of the Eyjafjallajökull eruption (May 22, 2010; Gudmundsson, *et al*.^59^), and biological and physico-chemical Fe removal (precipitation and scavenging). Patches with enhanced surface water DFe concentrations above 0.4 nM were encountered at two stations in the central IB and at one station near the south Greenland coast (Fig. 2c), which may be related to (sub-) mesoscale processes. Total dissolvable Fe concentrations were similarly patchy, showing locations with enhanced values (>1.5 nM) in the central IB and IRB as well as on the Greenland Shelf (Fig. 2d).

We compared average surface water DFe and TDFe concentrations between the IB and IRB, as well as between spring and summer (Table 1). We included nitrate to assess the consequences of biological uptake. Average DFe concentrations consistently represented about 14% of the TDFe pool for both seasons. In terms of the distributions of DFe, TDFe and nitrate, the two basins showed considerable differences for both seasons. In spring, DFe and TDFe were significantly higher in the IB compared with the IRB, by 2 fold and 3 fold, respectively, and nitrate lower by 1.4 fold (Table 1). This has been attributed to the enhanced volcanic ash inputs into the IB from the Eyjafjallajökull eruption, with the IRB being much less affected due to the transport of the ash predominantly in a southeasterly direction from the volcano over the IB^56,60^. In summer, DFe and TDFe concentrations were not significantly different between the two basins (IB DFe 0.099 ± 0.095 nM and IRB DFe 0.087 ± 0.234 nM). Nitrate was nearly 5 fold higher in the IRB (2.97 ± 1.57 µM) than in the IB (0.65 ± 0.76 µM), with the depleted levels in the IB explained by enhanced nitrate removal as a result of the ash-derived Fe inputs^56^ resulting in Fe and nitrate co-limited microbial communities in the IB^32^. In summer, both DFe and TDFe concentrations were significantly lower by 2.5 to 3 fold in the IB compared with spring, but the levels in the IRB were rather constant, most likely due to the relatively low spring values in the IRB due to the minimal ash inputs from Eyjafjallajökull volcano.

### Concentration profiles of DFe and TDFe

**Figure 3:**
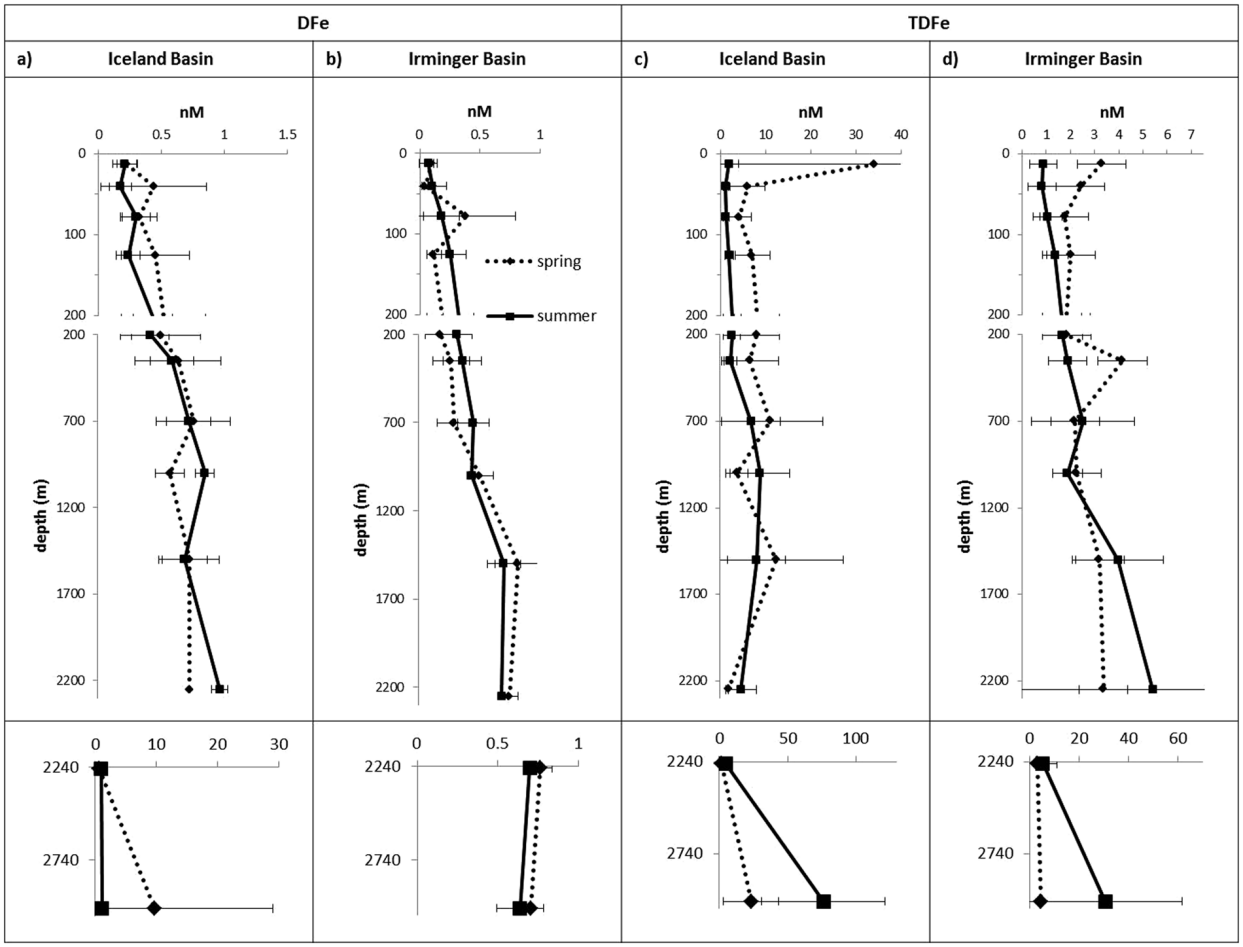
Average profiles (with SD of ±2σ) of DFe and TDFe in IB and IRB during spring and summer 2010, with bottom depths presented in separate box with expanded concentration range. Note the strongly increased and variable TDFe concentrations in the upper 200 m of the Iceland Basin during spring, with maximum TDFe concentration at the surface 34 ± 41 nM. Error bars represent the range of concentrations averaged at this depth interval (3–11 data points at each depth).

**Figure 4:**
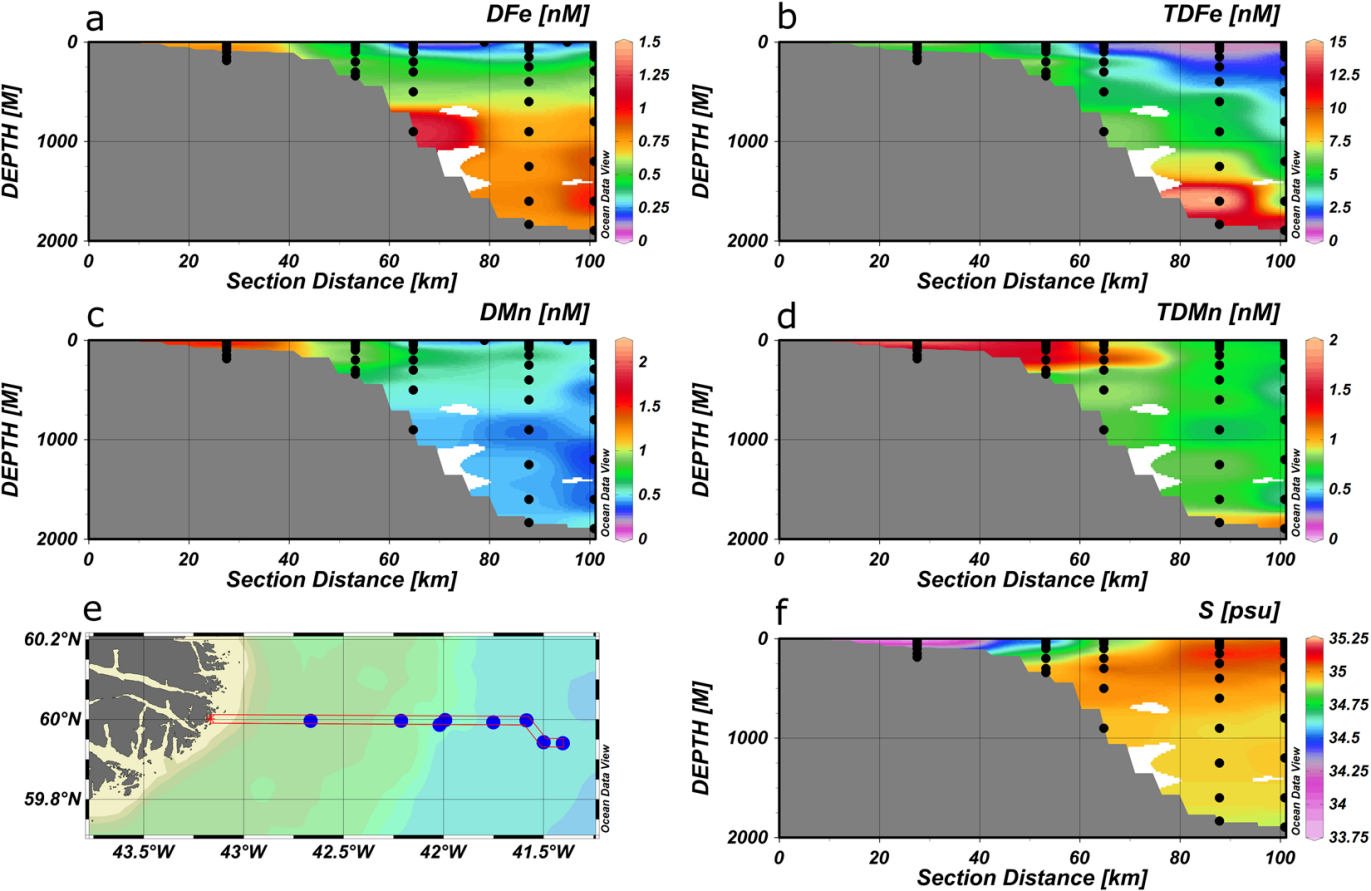
Section plots of DFe (**a**), TDFe (**b**), DMn (**c**), TDMn (**d**), salinity (**f**) on a transect along 60°N onto the Greenland shelf (**e**) with data from summer 2010. Figures were produced using Ocean Data View (Schlitzer, R., Ocean Data View, odv.awi.de, 2017).

**Figure 5:**
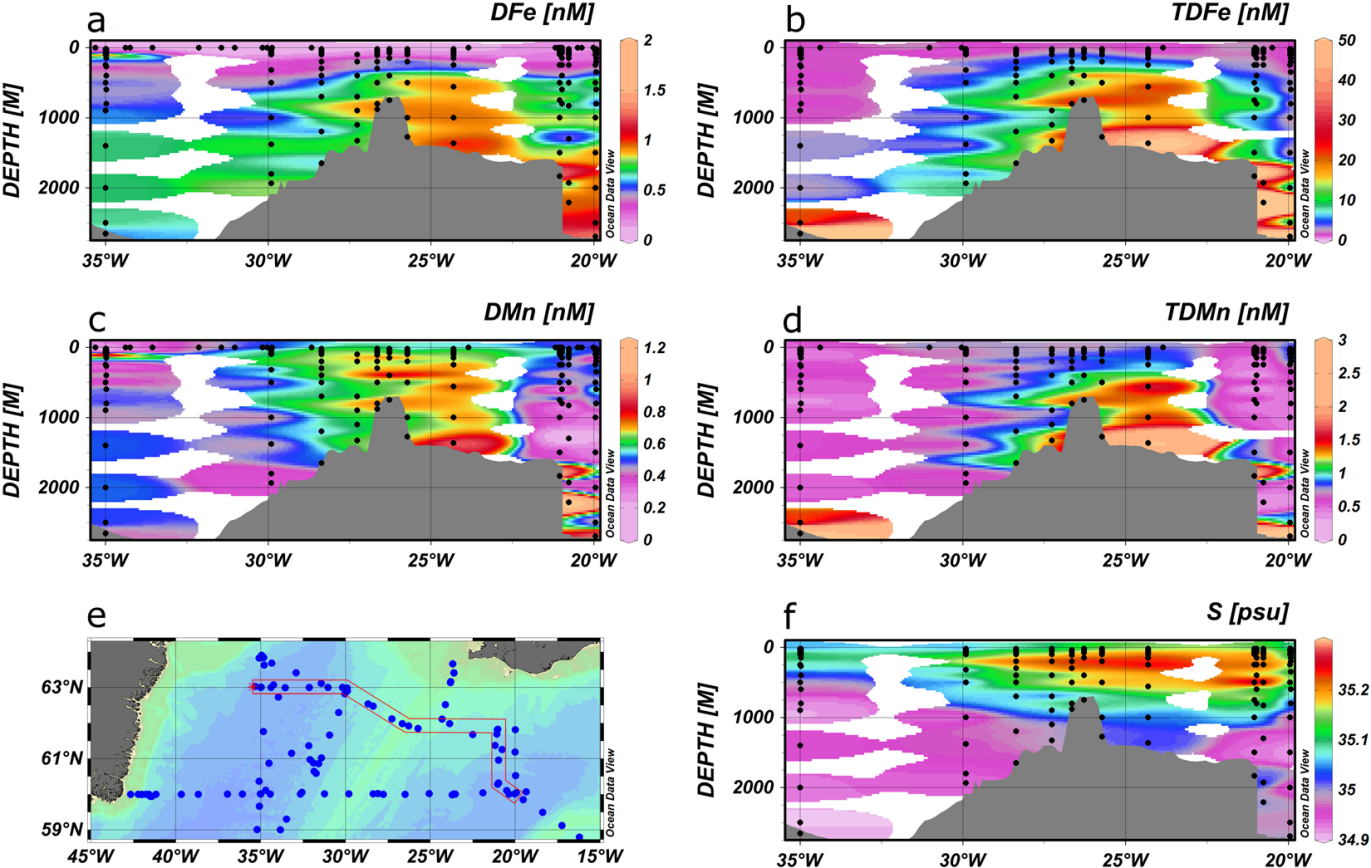
Section plots of DFe (**a**), TDFe (**b**), DMn (**c**), TDMn (**d**), salinity (**f**) on a transect across the Reykjanes Ridge at 62°N (**e**) with data from summer 2010. Figures were produced using Ocean Data View (Schlitzer, R., Ocean Data View, odv.awi.de, 2017).

**Figure 6:**
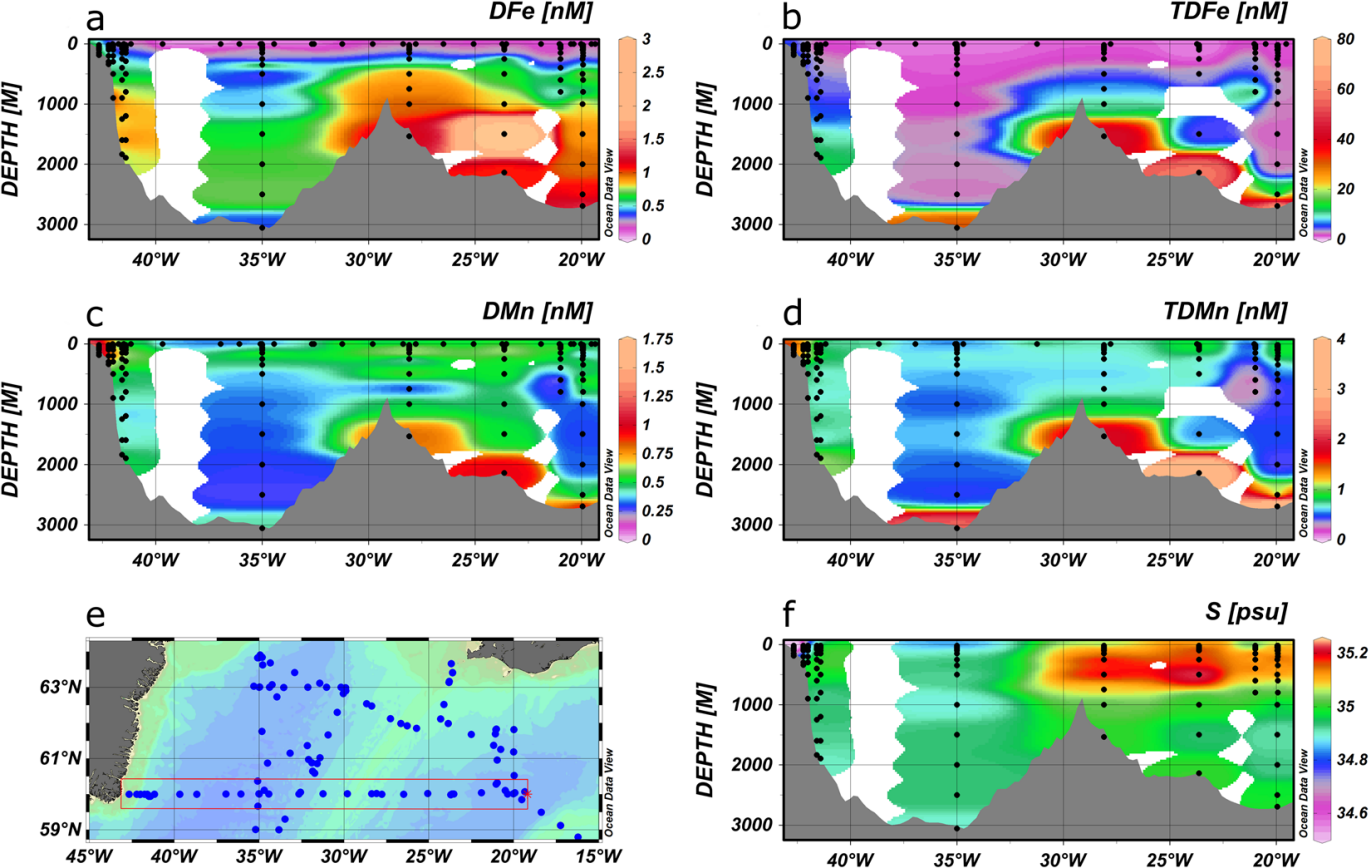
Section plots of DFe (**a**), TDFe (**b**), DMn (**c**), TDMn (**d**), salinity (**f**) on a transect along 60°N (**e**) with data from summer 2010. Figures were produced using Ocean Data View (Schlitzer, R., Ocean Data View, odv.awi.de, 2017).

We calculated average concentration profiles of DFe and TDFe (Fig. 3) for stations in each basin for each season, allowing us to compare the inter-basin and seasonal differences in the vertical structures and partitioning between the DFe and TDFe pools. Dissolved Fe showed the lowest concentrations (<0.3 nM) in the upper 100 m (Fig. 3a,b), consistent with its reported behaviour involving biological Fe uptake and particle scavenging in the surface waters^61^. At greater depths, the concentrations increased until ca. 1000 m, reaching values between 0.6–0.9 nM, likely to be partially a result of remineralisation of sinking particles, and then remained fairly constant below 1000 m with the exception of a maximum of about 10 nM observed near the seafloor in spring in the IB. High DFe concentrations near the seafloor most likely represents the influence of the nepheloid layer of resuspended sediments^62^ and further seafloor sources (see below), which is also reflected by the very high TDFe concentrations (up to 76 nM; Figs 4, 5 and 6) in that part of the water column.

Vertical profiles of TDFe showed enhanced concentrations in the upper 50 m of the water column during spring (50–150 times higher concentrations than DFe) likely due to the presence of volcanic ash particles (notably in the IB; Fig. 3c), in addition to biogenic particle formation in the course of the spring phytoplankton bloom (Fig. 3c,d). In summer the distribution of TDFe roughly followed the pattern of DFe profiles, with enhanced TDFe concentrations observed towards the sea floor (50–70 times higher concentrations than DFe) indicating accumulation of settling material from the spring bloom in both basins, in addition to sediment resuspension and seafloor sources (see below). In the upper 700 m of the IB, the TDFe concentrations were higher in spring compared with summer. This seasonal change in the IB is most likely due to ash inputs from the Eyafjallajokull eruption in spring 2010^56^ which was subsequently lost from the upper water column by summer. A sample of volcanic ash collected in the near-field volcanic plume^56^ had a mean grain size of 123 µm and would sink >700 m day^−1^, with only a small proportion (1.5% of bulk ash) featuring a grain size of ca. 20 µm with a sinking velocity of 0.2 mm s^−1^, thereby taking more than 1 month to sink to 700 m depth^56^. Interestingly, the average TDFe values in the IRB showed little difference between spring and summer, again likely the result of the minor impact of the volcanic eruption on the IRB. The TDFe concentrations in the depth profiles presented in Fig. 3c,d were generally about 4–17 times higher than DFe. Few studies report TDFe, but recent work in the Peru upwelling regime showed TDFe concentrations of up to 450 nM and DFe representing typically between 12 and 28% of the TDFe pool^63^, and hence similar to our observations.

The DFe concentrations did not show important variations with season or basin, with the exception of the enhanced surface ocean DFe values in the IB, particularly close to Iceland, in spring (see above). This likely reflects the dynamic equilibrium situation in the water column with DFe being buffered by natural organic ligands, and DFe in excess of ligand concentrations transferred to a readily exchangeable particulate pool through scavenging and precipitation processes^26,64–66^ which is then still in a dynamic exchange with DFe^21,67^.

### Ocean sections of DFe and TDFe

During summer 2010 we sampled three transects at a high spatial resolution. The first reached from 41.4°W along 60°N onto the Greenland shelf (Fig. 4). Enhanced surface DFe concentrations of 0.2 nM were observed along this section at ca. 60 km off the coast and increasing to >0.5 nM about 30 km off the coast (also see Fig. 6), which coincided with a drop in salinity indicating the presence of EGC waters with low temperatures (from 9.14 to 5.83 °C) and reduced salinities (from 34.99 to 33.84) due to melt water inputs. On the Greenland shelf (200 m depth) subsurface DFe concentrations reached >1 nM and were fairly constant below ca. 40 m depth, coinciding with enhanced DMn values (up to 1.48 nM) indicating a benthic source for these elements with release of soluble metal species following reductive dissolution^38^. The TDFe and TDMn concentrations were also elevated in the Greenland shelf waters and showed an increase near the sediments to values reaching 6.83 and 1.64 nM, respectively, due to sediment resuspension and/or scavenging of dissolved metals diffusing out of the sediments. It is likely that the sediment derived Fe inputs were augmented with Fe from melt waters as these form a source of DFe and particulate Fe (e.g. glacial flour) to the receiving waters, with an important proportion of the Fe in a bioavailable form^68,69^.

The second section was from 20 to 35°W and 60 to 63°N and crossed the Reykjanes Ridge at about 62°N (Fig. 5), and the third section was along 60°N, between ca. 19 to 43°W, crossing the Reykjanes Ridge at ca. 29°W (Fig. 6). The dynamic behaviours of Fe and Mn, with strong input and removal processes, coupled to the presence of relatively young watermasses in the high latitude North Atlantic^52^, meant that no distinct elemental signatures could be observed in any of the watermasses (Figs 5, 6).

The most striking feature along the sections is the enhanced concentrations of DFe (up to 1.03 nM), TDFe (up to 57 nM), DMn (up to 0.96 nM) and TDMn (up to 3.25 nM) over the Reykjanes Ridge. Our observations of elevated Fe and Mn signals may be attributed to two possible processes: enhanced turbulent mixing over the Reykjanes Ridge causing resuspension of sunken particles, and hydrothermal inputs. Mid-Ocean Ridges zones are regions of intensified deep mixing, and the mechanisms responsible for the deep turbulent mixing over the rough topography of the ridge systems and inside the numerous mid-ocean fracture zones are related to internal tides, near-inertial waves and mean flows^70,71^. Mid-ocean tidal velocities are weak with negligible turbulence generation on their own, but their interaction with rough topography can generate internal waves, which can propagate into the water column and generate enhanced turbulence to 1000–2000 m above the seafloor^72^. Furthermore, an important component of the NADW mean flow is southwards as part of a diffuse interior circulation south of the Denmark Strait^73^. The deep water speeds accelerate whilst passing through constrictions and moving across sills of the Reykjanes Ridge and become much greater than the tidal or overlying geostrophic flows, thereby causing enhanced turbulent mixing which may reach the thermocline waters^70^. This enhanced mixing over the Reykjanes Ridge may transfer sunken particulate material into the overlying water column, with associated dynamic exchange with DFe and DMn.

Hydrothermal activity along the Reykjanes Ridge may add to the dissolved and particulate Fe and Mn loads in the water column. This Ridge is a slow spreading system (10 mm y^−1^)^74^, and supports lower hydrothermal vent frequencies and venting activity compared to fast spreading ridges^75^, such as the Southeast Pacific Rise^76^. GEOSECS distributions of the magmatic tracer ^3^He in the high latitude Northwest Atlantic provide evidence for hydrothermal activity, with nevertheless much reduced maximum δ^3^He (up to δ^3^He 4%) values compared to the South Atlantic (up to δ^3^He 15%)^77^, and the South Pacific (over δ^3^He 35%^78^). A detailed survey for hydrothermal activity on the Reykjanes Ridge from 57°45′N to 63°09′N (covering 750 km ridge crest), including TDMn analyses, revealed only one site^74^ and raised the question whether the hot-spot affected ridges are systematically deficient in convective hydrothermal cooling^76^. The DFe to DMn ratios in deep waters over the Reykjanes Ridge ranged between 0.9 and 1.84. However, we assume that hydrothermally derived DFe is removed at a greater rate than DMn^79^, resulting in enhanced TDFe to TDMn ratios ranging between 5.7 and 16.5 over the Ridge. Reported endmember DFe to DMn ratios (unfiltered samples, but with typically no noticeable precipitates) at comparable basalt hosted hydrothermal systems are comparable or lower than our total dissolvable ratios: ca. 7–9 (Edmond)^79^, 4–7 (Kairei)^79^, ~6 (Beebe)^80^, ~8 (TAG)^81^. These endmembers would yield lower TDFe to TDMn ratios than we observed, following transport of vent fluids away from the vents. The GEOSECS ^3^He and our observations of the TDFe to TDMn ratios therefore indicate a relatively low contribution of hydrothermal inputs to deep ocean waters over the Reykjanes Ridge.

Recent large-scale observations in the Indian^82^, Pacific^76,83,84^ and Atlantic^39,66^ Oceans evidenced the horizontal transport of hydrothermal DFe over hundreds to thousands of kilometres. Stabilisation of hydrothermal DFe against precipitation, aggregation and scavenging loss upon entry into the oxic, cold and alkaline deep ocean waters is facilitated by organic ligands^85^ and particles^67,86^. Transport of hydrothermal DFe from the TAG sites (spreading rate ca. 20 mm y^−1^) in the North Atlantic reached >500 km^66^, in the South Atlantic >1000 km^39^, in the South East Pacific Rise (spreading rate >140 mm y^−1^) 4300 km^76^ and at our study site on the Reykjanes Ridge (spreading rate ca. 10 mm y^−1^) appeared to reach ca. 250–300 km (Figs 5,6). This comparison implies that the magnitude of the plume distribution may be linked to ridge spreading rate, which determines hydrothermal activity, with additional influences by deep ocean currents and ridge bathymetry^75^. The distributions of TDFe and TDMn followed similar distributions to the dissolved forms of these elements, with TDFe concentrations between 10 and 50 times higher than DFe in the plume, again suggestive of a dynamic equilibrium between the particulate and dissolved fractions^21,67^.

**Figure 7:**
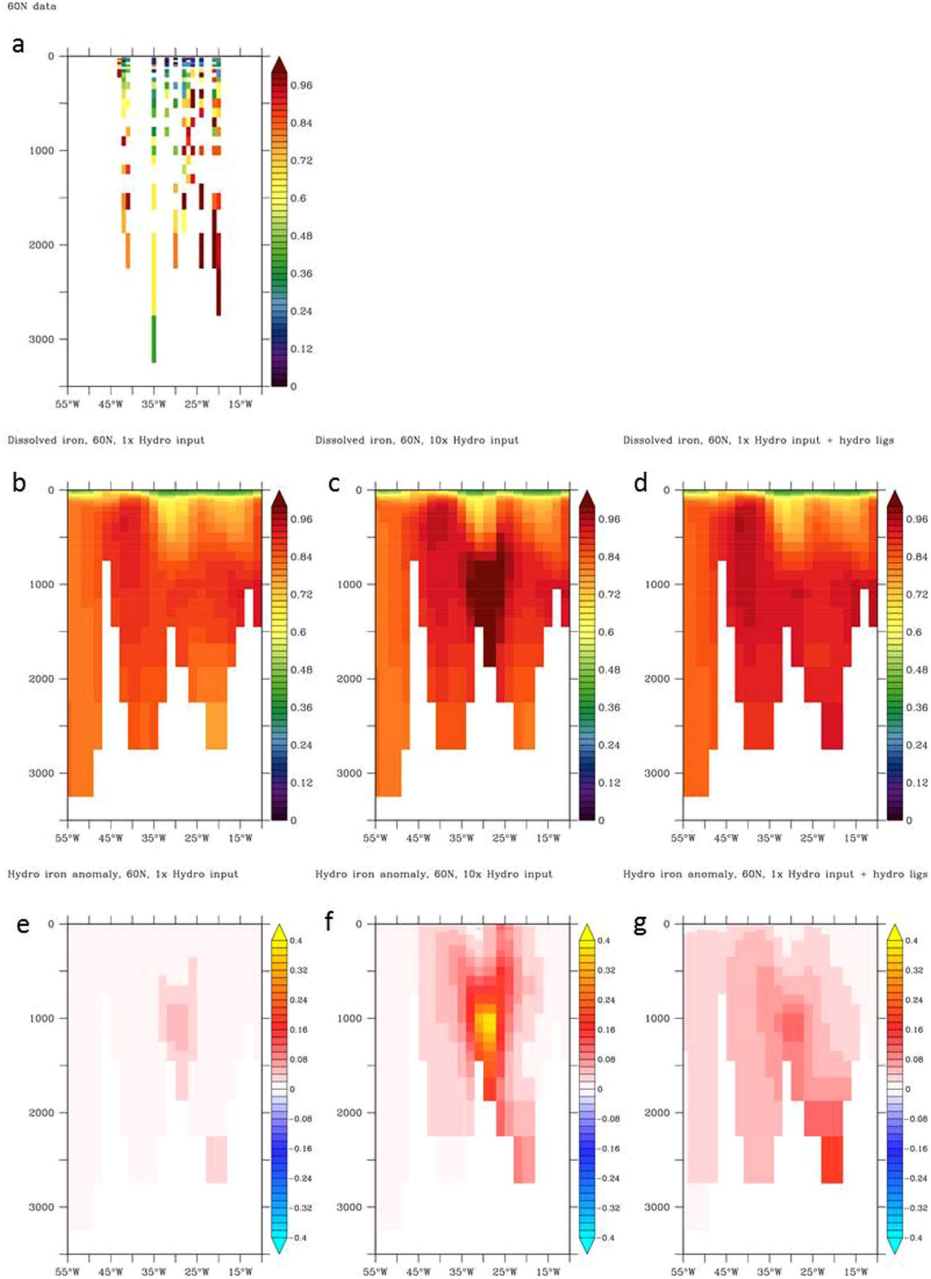
(**a**) Section plots of DFe observations along 60°N with data from summer 2010 with colour coding in nM; (**b**) Hydrothermal Fe supply according to a fixed DFe to ^3^He ratio^87^; (**c**) 10 times greater hydrothermal Fe supply compared to the fixed DFe to ^3^He ratio; (**d**) Hydrothermal Fe supply according to the fixed DFe to ^3^He ratio alongside an equimolar flux of iron-binding ligands; (**e**) Anomaly of hydrothermal Fe supply according to a fixed DFe to ^3^He ratio; (**f**) Anomaly of 10 times greater hydrothermal Fe supply compared to the fixed DFe to ^3^He ratio; (**g**) Anomaly of hydrothermal Fe supply according to the fixed DFe to ^3^He ratio alongside an equimolar flux of iron-binding ligands.

Initial work modelling hydrothermal Fe inputs used ^3^He inputs as a function of ridge spreading rate and a fixed DFe to ^3^He ratio estimated from a global compilation of hydrothermal fluids^87^, which indicated substantial variability. The 1 × 1 model grid requires the ‘effective’ flux at these spatial scales away from vents, and because observations remain scarce, the most efficient way of estimating the flux ratios is by testing different model scenarios against field data, as performed in this and previous^39,50,76^ studies. The pioneering modelling work worked well in reproducing data in the Southern Ocean but was found to underestimate the magnitude of hydrothermal anomalies over the slow spreading mid Atlantic ridge in the south Atlantic^39^ and the longevity of a hydrothermal plume from the fast spreading southern East Pacific Rise^76^. In agreement with previous work on the mid Atlantic ridge, comparing different modelling scenarios against this dataset (Fig. 7), suggests that a 10× greater hydrothermal Fe supply is necessary to improve the match to observed data (Fig. 7c), which may be partly influenced by the Fe resuspended through deep turbulent mixing. Hydrothermal Fe anomalies in the ocean interior are suggested to be on the order of 0.4 nM over the ridge (Fig. 7f). As seen on other GEOTRACES sections across the mid Atlantic ridge^50^, inclusion of hydrothermal Fe ligand supply is not as important as a greater gross hydrothermal Fe flux in reproducing Fe data (Fig. 7d,g).

The vertical distribution of DFe along the sections in the IRB revealed concentrations below 400 m depth of 0.6–0.8 nM, which agree with those reported for the GEOTRACES cruise GA02 of 0.6–0.8 nM^24^, but are lower compared to the IB and the Reykjanes Ridge region (ca. 1 nM) (also see Fig. 5). These observations are likely related to the enhanced contributions of seafloor and volcanic ash sources to the surveyed regions of the IB. The DMn distributions were similar to DFe in the deeper waters, but showed a contrasting surface water behaviour with relatively enhanced concentrations (ca. 0.6 nM), which is linked to photochemical reduction of Mn from particles^88^.

The influence of remineralisation of sinking particles on DFe distributions in subsurface waters (>40 m) can be estimated using the relationship with apparent oxygen utilisation (AOU; difference between observed dissolved oxygen concentration and its saturation concentration computed at the potential temperature of water and 1 atm total pressure)^45^, with exclusion of samples showing benthic DFe inputs as evidenced from enhanced DMn concentrations. For stations in the IB, the DFe:AOU relationship had a slope of 0.0087 ± 0.0012 nmol µmol^−1^ with an intercept of 0.29 ± 0.04 nM (R^2^ = 0.26, n = 146), whilst in the IRB the slope was 0.0150 ± 0.0010 nmol µmol^−1^ and intercept −0.03 ± 0.033 nM (R^2^ = 0.68, n = 88) (see Fig. S8). The inferred Fe:C ratio (C calculated using AOU:C ratio of 1.39^89^) was thus 12.1 ± 1.67 µmol mol^−1^ for the IB and 20.85 ± 1.39 µmol mol^−1^ for the IRB, with the higher Fe:C ratios in the IRB possibly explained by a lower productivity in this basin with ca. 50% lower POC fluxes compared to IB^90^. Our Fe:C ratios are in the range of those reported for the (sub-) tropical North Atlantic of 11 µmol mol^−1^ ^91^, 20 µmol mol^−1^ ^92^, 9.7–12.4 µmol mol^−1^ ^17^ and 3.3–30 µmol mol^−1^ ^45^. The ratios are however higher than those observed in the North Pacific (3–4 µmol mol^−1^, Sunda^93^) and Southern Ocean (~2 µmol mol^−1^, Sunda^93^). The enhanced ratios in our study region compared with the North Pacific and Southern Ocean can be ascribed to an overall higher Fe availability within this system resulting from relatively higher Fe inputs from atmospheric and seafloor sources. It should however be noted that only the DFe preserved in the dissolved phase following remineralisation can be combined with AOU to derive fluxes, which suggests these estimates will be conservative as some DFe associated with planktonic biomass should have been lost due to scavenging^94^.

### Iron supply to surface waters of the IB and IRB

The high latitude North Atlantic features seasonal Fe limitation of primary productivity, related to a limited Fe supply relative to nitrate^30,32^. The transfer of Fe to surface waters for the following sources has been estimated: (i) atmospheric inputs, (ii) winter convective supply, (iii) diffusive fluxes, (iv) horizontal fluxes.

### Atmospheric supply

Atmospheric inputs of Fe obtained from direct aerosol sampling during the spring and summer cruises are presented in Table 2. Rates of dry deposition of soluble Fe input during the spring cruise were highly variable and ranged from 2 to 2741 nmol m^−2^ d^−1^. Fluxes for samples not affected by the volcanic ash emissions (TM02 in IB, and TM03–04 in IRB) were similar to those of the summer cruise for the IB and IRB (TM10–15), and also 2007 and 2009 cruises in the region (2 to 88 nmol m^−2^ d^−1^ using v_d_ 0.7 cm s^−1^), while the highest fluxes (samples TM07-TM09) were associated with direct deposition of volcanic ash from the plume of the Eyjafjallajokull eruption (locations of aerosol sampling depicted on Fig. S4). Intermediate fluxes (samples TM05 & TM06 in IB) were above background levels, probably as a result of longer-range transport of ash from the eruption, as indicated by airmass backtrajectories (SI Figs S5–S7). The geographical extent of the direct volcanically-derived input flux was rather limited and restricted to waters immediately around Iceland^56,59^, but nevertheless the higher flux estimates must be treated as atypical.

**Table 2:** Atmospheric deposition rates of Fe during spring and summer 2010.

Station	Date	Mid Lat (^o^N)	Mid Lon (^o^W)	Aerosol soluble Fe concentration <1 µm (pmol m^−3^)	Aerosol soluble Fe concentration >1 µm or total^a^ (pmol m^−3^)	Dry Fe deposition (nmol m^−2^ d^−1^)	Notes/Citations
Spring 2010							Coincident with *Eyjafjallajokull* volcanic eruption 14^th^ April – 22^nd^ May
TM02	28/04/10	58.38	20.88	—	4 ± 0.3^a^	2^a^	
TM03	30/04/10	59.70	30.59	—	31.7 ± 6.8^a^	19^a^	
TM04	03/05/10	59.98	30.26	—	51.8 ± 7.1^a^	31^a^	
TM05	06/05/10	60.97	22.88	—	159.2 ± 3.6^a^	96^a^	
TM06	07/05/10	62.50	19.97	—	227.5 ± 5.3^a^	138^a^	
TM07	08/05/10	63.10	18.75	—	4532 ± 60. 5^a^	2741^a^	
TM08	08/05/10	63.09	19.08	—	3976 ± 85^a^	2405^a^	
TM09	09/05/10	63.39	21.31	—	351.3 ± 174.9^a^	212^a^	
**Summer 2010**							
TM10	18/07/10	60.00	38.59	3.7 ± 0.5	2.1 ± 1	2.2 ± 0.9^b^	42
TM11	20/07/10	60.72	38.49	3.2 ± 1.1	6.1 ± 1.5	5.6 ± 1.4^b^	42
TM12	24/07/10	61.19	32.98	2 ± 0.3	2.9 ± 0.5	2.7 ± 0.5^b^	42
TM13	27/07/10	59.42	33.40	6.3 ± 2.4	11.8 ± 1	10.8 ± 1.1^b^	42
TM14	31/08/10	63.41	29.43	5 ± 1.1	10 ± 0.7	9.0 ± 0.7^b^	42
TM15	05/08/10	61.18	22.42	4.2 ± 1.8	6.5 ± 0.6	5.9 ± 0.7^b^	42

^a^Reported aerosol concentration and dry depositional fluxes of Fe based upon total soluble Fe concentrations only. Dry deposition rate calculated assuming depositional velocities of 0.7 cm s^−1^ due to presence of volcanic ash in aerosol samples.

^b^Dry deposition rates calculated assuming depositional velocities of 1 cm s^−1^ for the coarse aerosol fraction (>1 µm) and 0.1 cm s^−1^ for the fine aerosol fraction (<1 µm).

The latitudes and longitudes are the midpoints of aerosol sample collection.

The size fractionated aerosol soluble Fe concentrations for the <1 µm and >1 µm fractions observed during the summer cruise were approximately equal. Rates of soluble Fe inputs during the summer were lower compared to spring, and in the summer the inputs were more consistent between samplings ranging from 2.2 to 10.8 nmol m^−2^ d^−1^ (Table 2). However, the majority of aerosol sampling was undertaken within the IRB and on average deposition rates here were 6.06 nmol m^−2^ d^−1^. However, the only transect to sample the IB produced an average flux of 5.9 nmol m^−2^ d^−1^ suggesting that a typical atmospheric Fe flux of ~6 nmol m^−2^ d^−1^ is not unusual for the study region, at least during summer 2010.

Modelled mean aerosol total Fe deposition rate estimates to our study region range from 49–490 nmol m^−2^ d^−1^^95^ and 340–860 nmol m^−2^ d^−1 12^. When considering an aerosol Fe solubility of 0.4% and 4%^96^, which are suggested solubilities for dust and combustion aerosols, respectively, we obtain soluble Fe fluxes of 0.20–1.96 nmol m^−2^ d^−1^ and 1.96–19.6 nmol m^−2^ d^−1^ for the deposition rates by Duce and Tindale^95^, and 1.36–3.44 nmol m^−2^ d^−1^ and 13.6–34.4 nmol m^−2^ d^−1^ for Jickells, *et al*.^12^. Our observations for soluble Fe are therefore in reasonable agreement with modelled literature values, whilst recognising that the literature estimates are annual and do not reflect the intra-annual variability of dust deposition in the high latitude North Atlantic that can be important^29^.

### Horizontal diffusive surface ocean fluxes of Fe

Potential sources for horizontal surface ocean Fe transport include continental shelf sediments and freshwater run-off^43,57^. In addition, we observed enhanced DFe concentrations at depth over the Reykjanes Ridge, which may be related to enhanced diffusive mixing over the ridge (e.g. Fig. S3a). We calculated surface water fluxes, and considered two main sources for the IB (Icelandic shelf and Reykjanes Ridge) and two main sources for the IRB (Greenland shelf and Reykjanes Ridge) (Figs S1–S3). We recognise that the estimates for diffusive horizontal flux are likely a lower bound, as the larger scale currents also play a role in transport of material.

The calculated DFe fluxes ranged from negligible (DFe gradient <0.00009 nM km^−1^) for north and southwards supply to the IRB, to 16.4 and 3.84 nmol m^−2^ d^−1^ from the Reykjanes Ridge into the IRB and IB, respectively, and 27.6 nmol m^−2^ d^−1^ from the Iceland shelf into the IB (Table 3). The TDFe gradients and fluxes for these regions were negligible (TDFe gradient <0.00009 nM km^−1^).

**Table 3:** Source and horizontal flux of dissolved Fe (DFe) and total dissolvable Fe (TDFe) in the Iceland and the Irminger Basins.

Source (nmol m^−2^ d^−1^)	Irminger Basin		Iceland Basin	
	DFe	TDFe	DFe	TDFe
Greenland Shelf	321*	9680*	—	—
Reykjanes Ridge	16.4	0	3.84	0
Iceland Shelf	—	—	27.6	nd

^*^DFe and TDFe fluxes from the Greenland Shelf to the Irminger Basin do not propagate further than the shelf itself. nd denotes not determined.

The highest fluxes were determined for the DFe and TDFe transfer from the Greenland shelf towards the IRB. However, these west to east transect gradients in the IRB (Transect A, Fig. S1) do not propagate further than ~100 km away from the coast (Figs 4, S3a,c) which is related to the strong density gradients along the eastern boundary of the EGC that carries Arctic water along the Greenland shelf (Figs 1 and 4). The Greenland shelf therefore does not constitute a major Fe source to the IRB. The uncertainties in the horizontal fluxes are large (see more detail in SI), and this is also indicated by the large difference between lower and upper horizontal Fe flux limits (Table 4).

**Table 4:** Summary of dissolved Fe (DFe) and total dissolvable Fe (TDFe) inputs to surface waters of Iceland and Irminger Basins (IB and IRB, respectively).

Source	Lower limit flux (in nmol m^−2^ d^−1^)	Upper limit flux (in nmol m^−2^ d^−1^)	Reference
Atmospheric soluble Fe	2.2	10.8 (2741^#^)	This study
Vertical DFe	−10.4	21.1	^42^
Horizontal DFe	0	Max 27.6 (321*)	This study
Horizontal TDFe	0	0 (9680*)	This study
Winter mixing DFe	27.3 ± 7.2 (IRB)	103 ± 40.9 (IB)	This study

The negative vertical flux involves DFe transfer out of surface mixed layer. Atmospheric soluble Fe inputs associated with the Eyjafjallajökull volcanic eruption are denoted by ^#^(upper limit). Horizontal fluxes from the Greenland Shelf (denoted by*) are prevented by strong density gradients in the East Greenland Current from reaching the Irminger Basin.

### Diffusive vertical fluxes

Diffusive Fe fluxes were determined at 21 stations during summer 2010. In general there was widespread variability in the direction and magnitude of diffusive Fe fluxes, and fluxes both into and out of the mixed layer were commonly observed. The high variability in diffusive Fe flux was a function of near-surface Fe gradients, and related to Fe removal by biological uptake and scavenging, and supply by organic matter remineralisation at the base of the mixed layer, as explained for this cruise by Painter *et al*.^42^. Consequently the overall picture of diffusive Fe supply to the subpolar gyre is rather inconsistent and no geographical or regional patterns could be discerned. It is clear however that the diffusive supply of Fe was a small supply term to the surface mixed layer. The mean flux of dFe into the mixed layer was 4.4 ± 6.2 nmol m^−2^ d^−1^, whereas the mean loss of dFe from the mixed layer was nearly equal at 2.5 ± 3.5 nmol m^−2^ d^−1^. A DFe net supply to the mixed layer of only 1.3 ± 6.1 nmol m^−2^ d^−1^ was obtained but due to the large standard deviation the flux term is indistinguishable from zero^42^.

### Convective supply

The winter convective supply of Fe to the surface waters of the North Atlantic subpolar gyre is poorly quantified. Nevertheless, using observations of DFe reported by Nielsdóttir, *et al*.^30^ for the IB, Forryan, *et al*.^97^ estimated an annual convective Fe input of between 13 and 17 μmol m^−2^ based on convective mixing to 600 m depth derived from Argo float observations. The present study covers a wider geographical region and using a similar technique to Forryan, *et al*.^97^, Painter, *et al*.^42^ estimated convective Fe inputs of 10 ± 4.5 μmol m^−2^ yr^−1^ (27.3 ± 7.2 nmol m^−2^ d^−1^) to surface waters of the IRB, and 37.5 ± 15 μmol m^−2^ yr^−1^ (102.7 ± 40.9 nmol m^−2^ d^−1^) to the IB during winter 2010. The almost 3 fold larger convective supply of Fe calculated for the IB, as compared with Forryan, *et al*.^97^, appears due to interannual variability in maximum winter mixing depths in the high latitude North Atlantic^98^ and changes in *in-situ* concentrations of DFe. Specifically, winter mixing across the subpolar gyre in 2010 was relatively shallow and in the IB and IRB reached only to depths of 350 m and 200 m, respectively. The *in-situ* concentrations of DFe were also rather different between the two studies, with Forryan, *et al*.^97^ reporting a concentration of 0.42 nM at 600 m in the IB, whereas this study observed a concentration of 0.75 nM at 350 m. Consequently, the convective supply is somewhat larger than previous estimates despite the shallower convective mixing depth.

The observed atmospheric inputs, and diffusive vertical and horizontal fluxes are obviously subject to spatial and temporal variability and have an inherent uncertainty, but overall indicate that these fluxes of DFe into the IB and IRB are significantly lower than the fluxes associated with winter mixing which provide up to ca. 100 nmol m^−2^ d^−1^ and represent the largest single input term (Table 4). Hydrothermal inputs in addition to deep turbulent mixing affect subsurface Fe concentrations in the Reykjanes Ridge region and western IB, and will consequently have contributed to the enhanced convective fluxes.

The convective Fe fluxes directly determine the magnitude and duration of the spring bloom^97^, and any significant interannual variability in the magnitude of this convective Fe supply will have important implications for biological productivity in this region and perhaps on the prevalence and extent of Fe limitation during summer months^30,32^. Whilst low in magnitude (Table 4), the atmospheric, and diffusive horizontal and vertical fluxes provide a sustained Fe supply during the summer period after the winter mixing derived Fe has been exhausted. These supplies are however insufficient to alleviate the seasonal Fe limitation in the region, which ultimately results in the low ratios of Fe to nitrate in subsurface source waters^30,42^.

The anomolous high aerosol Fe inputs associated with the Eyjafjallajökull volcanic eruption (Table 4) were higher than any other source for the IB, resulting in biogeochemical consequences involving the development of Fe and nitrate co-limitation^32^. The exact timing of such volcanic inputs, in relation to the seasonal cycle of phytoplankton blooms and deep winter mixing, is important. A three times higher stimulation of biological productivity can be achieved, according to model simulations, when this aerosol Fe supply occurs later in the growing season^99^.

The maximum winter mixing depths along 60°N do not typically exceed 300 m over the mid Atlantic ridge according to De Boyer Montegut climatology^83^, which agrees with mooring observations in the central IB of depths down to 400 m^98^ but with excursions reaching 1 km during cold winters. Our observations (Fig. 6) indicate evidence of hydrothermal derived Fe at depths below 500 m over the mid Atlantic ridge, with the model indicating an Fe anomaly below ca. 700 m (Fig. 7). A potential contribution of the hydrothermal Fe to surface water primary production through convective supply is hence limited to seasons following cold winters.

## Electronic supplementary material

Supplementary Information
