# Supplementary material for: Iron Biogeochemistry in the High Latitude North Atlantic Ocean
Source: Sci Rep. 2018 Jan 19;8:1283. doi: 10.1038/s41598-018-19472-1 (PMC5775377; doi:10.1038/s41598-018-19472-1)
Supplement: Supplementary file 1 — Supplementary Information [file 41598_2018_19472_MOESM1_ESM.pdf]

Supplementary Information

Iron Biogeochemistry in the High Latitude North Atlantic Ocean

Eric P. Achterberg[1,2], Sebastian Steigenberger[1,3], Chris M. Marsay[1,5], Frédéric A. C. LeMoigne[2,3], Stuart C. Painter[3], Alex R. Baker[4], Douglas P. Connelly[3], C. Mark Moore[1], Alessandro Tagliabue[6], Toste Tanhua[2]

Sample collection

Samples were collected using trace metal clean 10 L OTE (Ocean Test Equipment) samplers mounted on a Ti CTD rosette frame. The OTE bottles were immediately transferred into a pressurized clean container (class 1000) for sub-sampling. In addition, surface seawater (~3 m depth) was pumped from a tow fish into the clean container using a Teflon diaphragm pump (Almatec A15) connected to a clean oil-free air compressor (JunAir) [1] and samples taken every two hours whilst the ship was in transit.

Samples for trace metal analysis were collected in acid-cleaned 125 or 250 ml low-density polyethylene bottles (LDPE, Nalgene) after on-line filtration with a 0.2 µm Sartobran 300 (with 0.4 µm pre-filter, Sartorius) cartridge filter. The samples from the OTE bottles were filtered under pure N_2 pressure (filtered 99.99% N_2, 1 bar), whereas those from the towfish were filtered using the pressure from the Teflon pump. Additional samples were collected without filtration. Each sample was acidified to pH ~1.9 on board in a laminar flow hood (class 100) with nitric acid (UpA HNO_3, Romil, UK) and stored in double bags until analysis at the National Oceanography Centre Southampton. The storage of the acidified (pH ~1.9) unfiltered samples with subsequent analysis after 18 months yielded total dissolvable Fe (TDFe) and manganese (TDMn) concentrations, which include dissolved (0.2 µm filtered) and an acid leachable fraction of the particulate pool. In order to

verify that sampling and sample handling procedures were trace metal clean, samples were analysed for DFe at sea using a flow injection technique [2].

Trace metal and nutrient determination

Concentrations of Fe were determined by isotope dilution inductively coupled mass spectrometry (ID-ICP-MS), and the mono-isotopic element Mn was analysed using a standard addition approach followed by ICP-MS detection, following the methods described in Milne, et al. [3]. The analysis was conducted following an off-line preconcentration/matrix removal step [3] on a WAKO chelate resin column[4]. Briefly, in an acid cleaned 30 mL FEP bottle (Nalgene) 12 mL of the acidified sample was spiked with a standard solution containing the stable isotope ^{57}Fe (ISOFLEX, USA) which was enriched over its natural abundance. The exact concentration of the isotopically enriched Fe in this standard solution was determined by ICP optical emission spectroscopy. In addition, standard additions of Mn were performed on sub-sets of seawater samples. After overnight equilibration the spiked samples were UV irradiated for at least 2 h and subsequently adjusted to pH 6.3 using ammonium acetate buffer, made from ultrapure acetic acid and ammonia (UpA, Romil, UK). The buffered sample was then pumped over the pre-concentration column, the column was rinsed with ultra high-purity water (UHP; MQ, Millipore, >18 MOhm cm^{-1}) to remove salts, and subsequently the metals were eluted with 1 mL of 1 mol L^{-1} HNO_3 (UPA; Romil, UK) and collected into acid cleaned polypropylene vials (4 mL; OmniVials) and capped. The pre-concentrated samples were analysed for ^{56}Fe, ^{57}Fe and ^{55}Mn using an Element II XR high resolution ICP-MS (ThermoFisher Scientific, Germany). The samples were introduced via a 100 µl Teflon nebuliser connected to a quartz spray chamber. Measurements for Fe and Mn were performed in medium resolution (R=4000). The Fe concentration in the sample was calculated using a standard isotope dilution equation [5,6]. The samples were corrected for buffer and the system (resin and ICP-MS) blanks, which were 13 pM Fe as buffer blank and about 5% of the sample signal counts per second (cps) as

system blank determined by introducing and analyzing elution acid (1 M HNO_3). The accuracy and precision of the method was assessed by analyses of SAFe (Sampling and Analysis of iron) reference samples (http://www.geotraces.org/science/intercalibration/322-standards-and-reference-materials). The values determined using the ID-ICP-MS method showed good consistency with the reported consensus values for DFe and DMn (Table 1). Dissolved Mn detected by ICP-MS yielded higher concentrations than the consensus values, in agreement with other observations that showed higher DMn concentrations obtained using ICP-MS analysis compared with catalytic-enhanced flow injection (http://www.geotraces.org/images/stories/documents/intercalibration/Files/Reference_Samples_November11/SAFe_Ref_Mn.pdf).

Nitrate, silicate and phosphate were analysed at sea using standard auto-analyser techniques [7] on a Skalar instrument, following the best practice guide for performing nutrient measurements at sea [8].

Aerosol sampling and analysis

During the spring, aerosol samples were collected onto single 20 x 25 cm Whatman 41 filters using a high volume aerosol collector (Tisch TSP) operating at a flow rate of ca. 1 m^3 min^{-1} [9] and located on the deck above the bridge of RRS *Discovery*. Collection filters were acid-washed before use, with 0.5 M HCl (Aristar Grade, Fisher), followed by 0.1 M HCl (Aristar Grade, Fisher). Separation of particles into aerodynamic diameters greater than or less than 1 μm was achieved during the summer cruise using a Sierra-type cascade impactor. Collection times for each sample were relatively long (2-3 days) because of very low aerosol concentrations in the study region, and the collector was only used when the ship was heading into the prevailing wind in order to avoid contamination from the ship.

After collection, samples were sealed in plastic bags and immediately frozen at -20°C for return to the land-based laboratory. Trace elements were leached from the filters using a 1 M ammonium

acetate solution (pH 4.7) and filtered through 0.2 µm filters (Minisart, Sartorius), as described in [9]. Soluble Fe was analysed by ICP-OES and here we report on the dry deposition fluxes of soluble Fe (F_{dry}, nmol m^{-2} d^{-1}) as the product of their aerosol concentrations (C_{aero}, pmol m^{-3}) and a dry deposition velocity (v_d).

$$F_{dry} = C_{aero}\, v_d$$

Values of v_d were set to 1 and 0.1 cm s^{-1} for the coarse (> 1 µm) and fine (< 1 µm) aerosol modes, respectively (summer cruise), and 0.7 cm s^{-1} for the spring cruises. Deposition velocities vary strongly as a function of particle size and wind speed [10] and are highly uncertain. Values of vd were set to 1 and 0.1 cm s^{-1} for the coarse (> 1 µm) and fine (< 1 µm) aerosol modes during the summer cruise[11]. For the spring cruise, for which size-segregated aerosol concentrations were not available, a single v_d value of 0.7 cm s^{-1} was applied. This value takes into account the distribution of soluble Fe between coarse and fine mode aerosols (as observed in the summer cruise). Samples containing high concentrations of volcanic ash during the summer cruise might have contained a higher proportion of coarse mode soluble Fe (although this cannot be confirmed), in which case dry deposition fluxes for these samples could be considered as lower limits. In any case, the use of dry deposition velocities to estimate fluxes is generally considered to be uncertain by a factor of 2 – 3 [11].

Aerosol Fe solubility is likely to be strongly influenced by the properties of the leaching solution used, as well as those of the aerosols [12]. Seawater properties (e.g. the presence and nature of strong Fe-binding ligands) are more variable than those of the simple leach used here, but over broad scales Fe solubility in seawater appears to be similar to that in ammonium acetate [13].

Diffusive flux measurements and calculations

Turbulent kinetic energy dissipation (ε) was measured using a free-fall microstructure shear profiler (MSS90L, Sea and Sun Technology GmbH and ISW Wassermesstechnik). The rate of turbulent kinetic energy dissipation was calculated from the variance of the measured vertical microstructure

shear by integration of the vertical microstructure shear power spectrum following the method of Forryan et al. (2012) [14] and assuming isotropic turbulence[15]. Turbulent diffusivity (K) was calculated from turbulent kinetic energy dissipation; full details of the approach are provided in Painter, et al. [16]. The top 8 m of data in the water column were omitted in order to remove near-surface influences prior to binning the diffusivity data into 4 m depth bins. Vertical diffusive fluxes of DFe were subsequently estimated from the turbulent diffusivity and profiles of DFe.

Horizontal flux calculations

Horizontal surface ocean Fe fluxes were calculated for both the IB and IRB following previous work [17-19]. The Fe fluxes have been estimated from the gradients of DFe and TDFe observed along transects from shelf regions to the open ocean and over the Reykjanes Ridge (transects A, B, C; Fig. S1) to the waters of the IB and IRB, using reported estimates of horizontal diffusivity. In the HLNA, diffusivities were determined from drifters and the Parallel Ocean Program model that includes eddy-induced mixing, and were up to ca. 1×10^7 cm^2 s^{-1} in both North/South and West/East components [20]. The uncertainties on the chosen diffusivity (1×10^7 cm^2 s^{-1}) is likely factor 2-3, and as the number of datapoints available to calculate the DFe gradients were limited (Fig. S2, S3), these gradients also have a large uncertainty.

Supply of Fe to the Iceland Basin- To determine the DFe flux from the Icelandic shelf to the IB (63.5ºN to 60.0ºN along 20ºW; ~350 km distance; transect C in Fig. S1), 21 samples were used (including tow fish and Ti CTD samples) from the surface waters and MLD (0-40 m). For DFe along transect C (Fig. S2a) we obtained the following relationship: $DFe_{surface} = -0.0028 \times distance + 1.045$, $r^2 = 0.53$, yielding an offshore DFe gradient of 0.0028 nM km^{-1}. The product of the gradient and the upper value of the diffusivity (1×10^7 cm^2 s^{-1}) yields a flux of DFe of 242 µmol m^{-2} d^{-1}. Assuming a summer MLD of 40 m and a 200 km long shelf supplying the IB, the flux of DFe from the Icelandic shelf is 1.94×10^3 mol d^{-1}. Normalisation to the length of the gradient and length of the shelf

provides a horizontal DFe flux to the IB of 27.6 nmol m^{-2} d^{-1}. No TDFe data was available for transect C.

The DFe flux from the Reykjanes Ridge to the IB was obtained using three surface datapoints from transect A between 29.8ºW to 23.7 ºW along 60ºN (~360 km, Fig. S3(a)) yielding *DFe* = -0.0004 x *distance* + 0.2113, r^2 = 0.62. This provides an offshore DFe gradient of 0.0004 nM km^{-1}, a flux of 0.69 x 10^3 mol d^{-1} considering a shelf length of 500 km and a MLD of 40 m, with a horizontal DFe flux of 3.84 nmol m^{-2} d^{-1} to the IB. The flux of TDFe was deemed negligible based on the absence of enhanced TDFe concentration in surface waters on the Reykjanes Ridge (Fig. S3c).

Supply of Fe to the Irminger Basin- No clear Fe gradients (in any direction) could be observed on transect B (Fig. S3b,d), and we therefore assume that the northwards or southwards DFe or TDFe fluxes in the IRB were negligible. On transect A, an eastward and westward gradient in DFe was observed (Figure 6a) indicating supplies by both the Greenland shelf and the Reykjanes Ridge to the IRB.

We took 18 points from -43.0ºW to -41.2 ºW (~70 km) from the surface DFe along transect A (Fig. S2b) to calculate the input of DFe from the Greenland shelf to the IRB. The relationship yields $DFe_{surface}$ = -0.0065 x *distance* + 0.4298, r^2 = 0.62, giving an offshore DFe gradient of 0.0065 nM km^{-1}. The product of this gradient and the diffusivity provides a flux of DFe of 0.562 x 10^3 µmol m^{-2} d^{-1}. Assuming a summer MLD of 40 m [16] and that the length of the Greenland shelf fuelling the IRB is 700 km, the flux of DFe from the Greenland shelf is 1.55 x 10^3 mol d^{-1}. Normalisation to the surface area influenced by the shelf (length of Greenland shelf x length of the gradient; ~700 km x ~70 km) provides a final horizontal DFe flux of 321 nmol m^{-2} d^{-1} supplied to the IRB. In the case of TDFe, we took two points between -42.0ºW to -41.2 ºW (~45 km; Fig. S3c) and the relationship yields $TDFe_{surface}$ = -0.1259 x *distance* + 5.6, with an offshore TDFe gradient of 0.1259 nM km^{-1}.

Using the same geographical assumptions as for DFe, the horizontal flux of TDFe is 9680 nmol m^{-2} d^{-1}.

The westward flux of DFe from the Reykjanes Ridge to the IRB was calculated by taking three datapoints on transet A from -29.8ºW to -32.6 ºW (~190 km; Fig. S3a) yielding $DFe_{surface}$ = -0.0009 x *distance* + 0.22399, r^2 = 0.91 giving an offshore DFe gradient of 0.0009 nM km^{-1}. This provides a final horizontal DFe flux of 16.4 nmol m^{-2} d^{-1} supplied to the Irminger basin from the Reykjanes Ridge, which is ca. 4 times larger than the flux from the Ridge to the IB. We assumed that the flux of TDFe was negligible based on the absence of enhanced TDFe concentration over the Reykjanes Ridge.

Winter Convective fluxes

Winter convective fluxes were obtained from examinations of individual Argo float data from the IRB and IB in winter 2010, which allowed determination of the mixed layer depths using criteria by de Boyer Montégut, et al. [21]. The obtained winter mixed layer depths were 200 m for the IRB and 350 m for the IB. Full details provided in [16]. Dissolved Fe concentrations from profiles were then integrated to the depth of winter mixing to provide an estimate of convective inputs as reported by (Nielsdottir et al. 2009)[22].

References

1 Achterberg, E. P. *et al.* Determination of iron in seawater. *Analytica Chimica Acta* **442**, 1-14, doi:10.1016/s0003-2670(01)01091-1 (2001).

2 Obata, H., Karatani, H. & Nakayama, E. Automated determination of iron in seawater by chelating resin concentration and chemiluminescence detection. *Analytical Chemistry* **65**, 1524-1528 (1993).

3 Milne, A., Landing, W., Bizimis, M. & Morton, P. Determination of Mn, Fe, Co, Ni, Cu, Zn, Cd and Pb in seawater using high resolution magnetic sector inductively coupled mass spectrometry (HR-ICP-MS). *Analytica Chimica Acta* **665**, 200-207, doi:http://dx.doi.org/10.1016/j.aca.2010.03.027 (2010).

4 Kagaya, S. *et al.* A solid phase extraction using a chelate resin immobilizing carboxymethylated pentaethylenehexamine for separation and preconcentration of trace elements in water samples. *Talanta* **79**, 146-152, doi:https://doi.org/10.1016/j.talanta.2009.03.016 (2009).

5 Heumann, K. G. Isotope-Dilution Mass-Spectrometry (Idms) of the Elements. *Mass Spectrometry Reviews* **11**, 41-67 (1992).

6 Heumann, K. G. Isotope-dilution ICP–MS for trace element determination and speciation: from a reference method to a routine method? *Anal. Bioanal. Chem.* **378**, 318-329, doi:10.1007/s00216-003-2325-z (2004).

7 Grasshoff, K., Ehrhardt, M. & Kremling, K. *Methods of Seawater Analysis*. (Verlag Chemie, 1983).

8 Hydes, D. J. *et al.* Determination of dissolved nutrients (N, P, SI) in seawater with high precision and inter-comparability using gas-segmented continuous flow analysers. (2010).

9 Baker, A. R. *et al.* Dry and wet deposition of nutrients from the tropical Atlantic atmosphere: Links to primary productivity and nitrogen fixation. *Deep-Sea Research Part I-Oceanographic Research Papers* **54**, 1704-1720 (2007).

10 Ganzeveld, L., Lelieveld, J. & Roelofs, G.-J. A dry deposition parameterization for sulfur oxides in a chemistry and general circulation model. *Journal of Geophysical Research: Atmospheres* **103**, 5679-5694, doi:10.1029/97JD03077 (1998).

11 Duce, R. A. *et al.* The atmospheric input of trace species to the world ocean. *Global Biogeochemical Cycles* **5**, 193-259, doi:10.1029/91GB01778 (1991).

12 Baker, A. R. & Croot, P. L. Atmospheric and marine controls on aerosol iron solubility in seawater. *Marine Chemistry* **120**, 4-13 (2010).

13 Baker, A. R., Laskina, O. & Grassian, V. H. in *Mineral Dust: A key player in the Earth System* (eds P. Knippertz & J.B. Stuut) 75-92 (Springer, 2014).

14 Forryan, A. *et al.* Turbulent nutrient fluxes in the Iceland Basin. *Deep Sea Research Part I: Oceanographic Research Papers* **63**, 20-35, doi:http://dx.doi.org/10.1016/j.dsr.2011.12.006 (2012).

15 Yamazaki, H. & Osborn, T. Dissipation estimates for stratified turbulence. *Journal of Geophysical Research: Oceans* **95**, 9739-9744, doi:10.1029/JC095iC06p09739 (1990).

16 Painter, S. C. *et al.* An assessment of the vertical diffusive flux of iron and other nutrients to the surface waters of the subpolar North Atlantic Ocean. *Biogeosciences* **11**, 2113-2130, doi:10.5194/bg-11-2113-2014 (2014).

17 Planquette, H. *et al.* Dissolved iron in the vicinity of the Crozet Islands, Southern Ocean. *Deep Sea Research part II* **57**, 1999-2019, doi:10.1016/j.dsr2.2007.056.019 (2007).

18 Bucciarelli, E., Blain, S. & Treguer, P. Iron and manganese in the wake of the Kerguelen Islands (Southern Ocean). *Marine Chemistry* **73**, 21-36 (2001).

19 Rijkenberg, M. J. A. *et al.* Fluxes and distribution of dissolved iron in the eastern (sub-) tropical North Atlantic Ocean. *Global Biogeochemical Cycles* **26**, doi:doi:10.1029/2011GB004264 (2012).

20 McClean, J. L., Poulain, P.-M., Pelton, J. W. & Maltrud, M. E. Eulerian and Lagrangian Statistics from Surface Drifters and a High-Resolution POP Simulation in the North Atlantic. *Journal of Physical Oceanography* **32**, 2472-2491, doi:doi:10.1175/1520-0485-32.9.2472 (2002).

21 de Boyer Montégut, C., Madec, G., Fischer, A. S., Lazar, A. & Iudicone, D. Mixed layer depth over the global ocean: An examination of profile data and a profile-based climatology. *Journal of Geophysical Research: Oceans* **109**, n/a-n/a, doi:10.1029/2004JC002378 (2004).

22 Nielsdóttir, M. C., Moore, C. M., Sanders, R., Hinz, D. J. & Achterberg, E. P. Iron limitation of the postbloom phytoplankton communities in the Iceland Basin. *Global Biogeochem. Cycles* **23**, GB3001, doi:10.1029/2008gb003410 (2009).

Table S1: SAFe reference seawater results obtained using ICPMS, along with reported community consensus values ((http://www.geotraces.org/science/intercalibration/322-standards-and-reference-materials).

	Fe (pM)	Mn (nM)
SAFe S-171	114±14	0.81±0.02
Consensus value S	93±8	0.79±0.06
SAFe D2-441	886±22	0.42±0.04
Consensus value D2	933±23	0.35±0.05

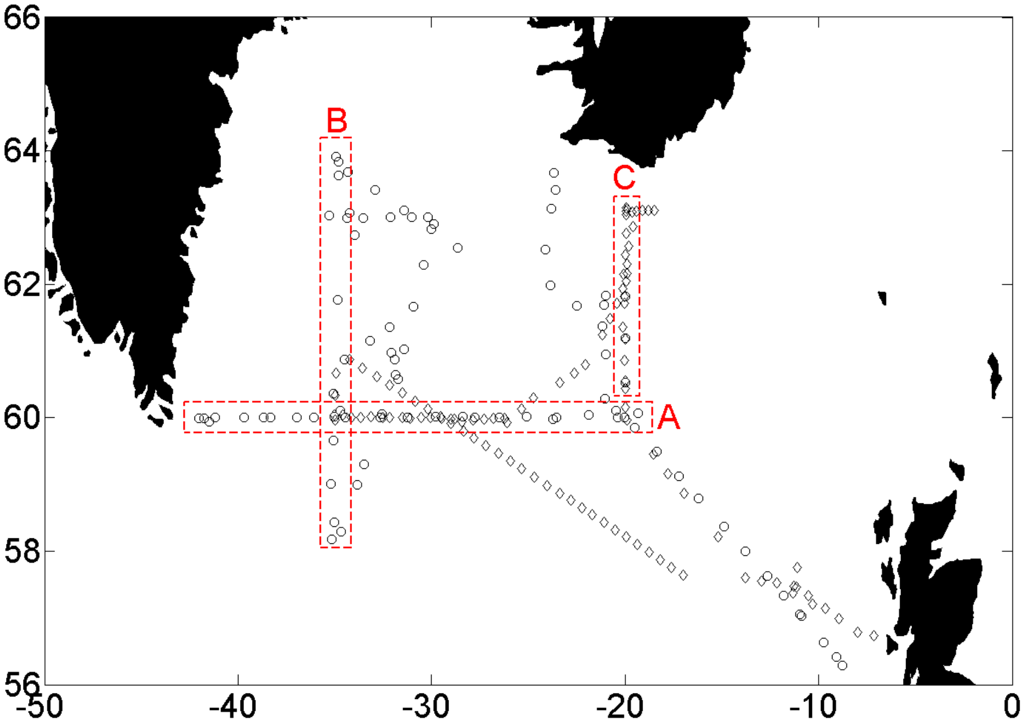

Figure S1: Sampling area of D354 during July and August 2010. Transects A, B and C are marked in red. Figure was produced with the software programme MATLAB (MathWorks®; https://www.mathworks.com) version 2011 using the toolbox "fillmap", which was produced in April 1995 by Matthew Jones and modified in December 1999 by Helen Snaith.

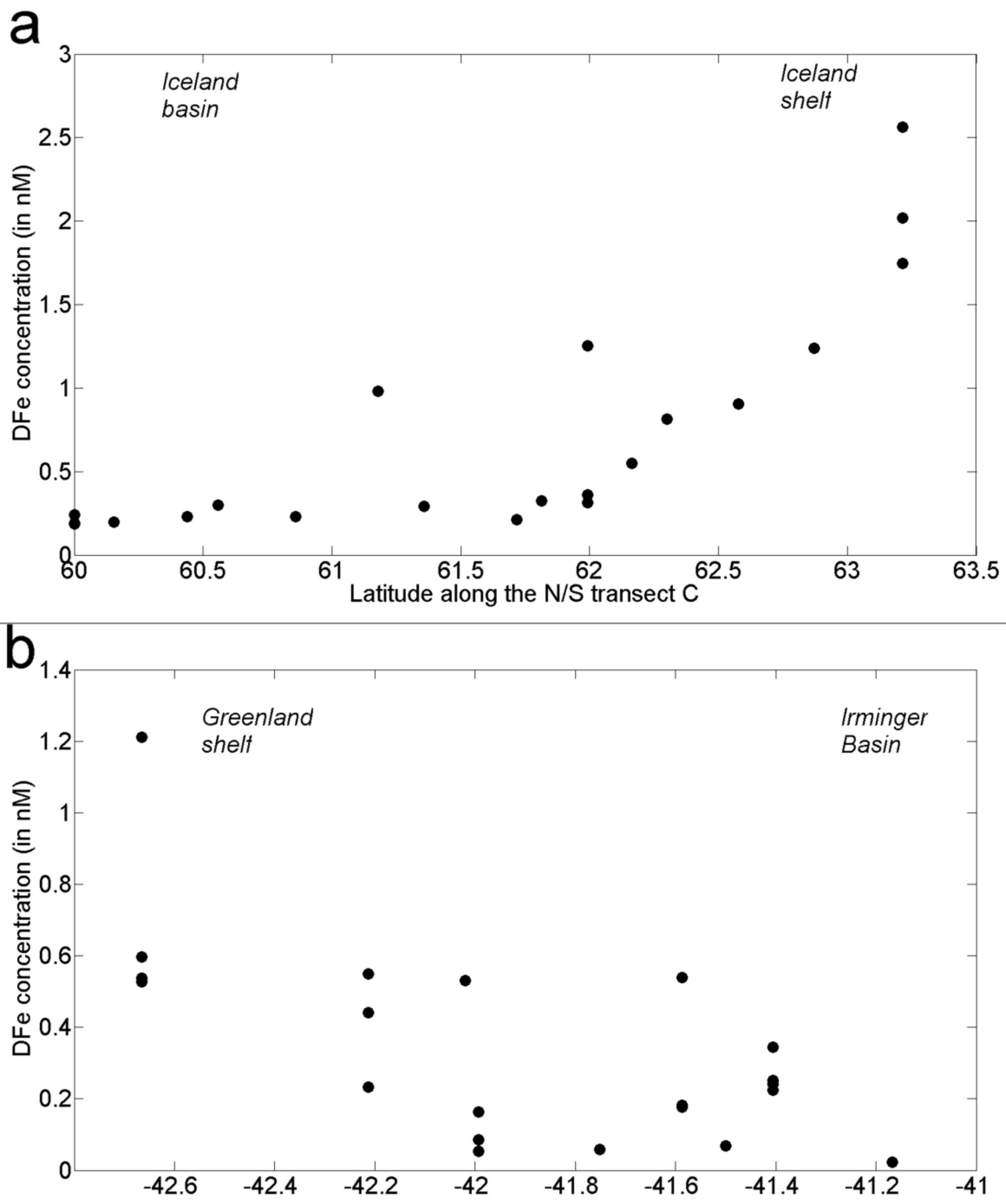

Figure S2: (a) Surface underway DFe concentrations (in nM) along transect C (N/S). Converted to km, this distribution yields to a relationship of *DFe* = -0.0028 x *distance* + 1.045, r^2 = 0.53, n=21. (b) Surface underway DFe concentrations (in nM) along transect A (W/E). Converted to km, this distribution yields to a relationship of $DFe_{surface}$ = -0.0065 x *distance* + 0.4298, r^2 = 0.62, n=18.

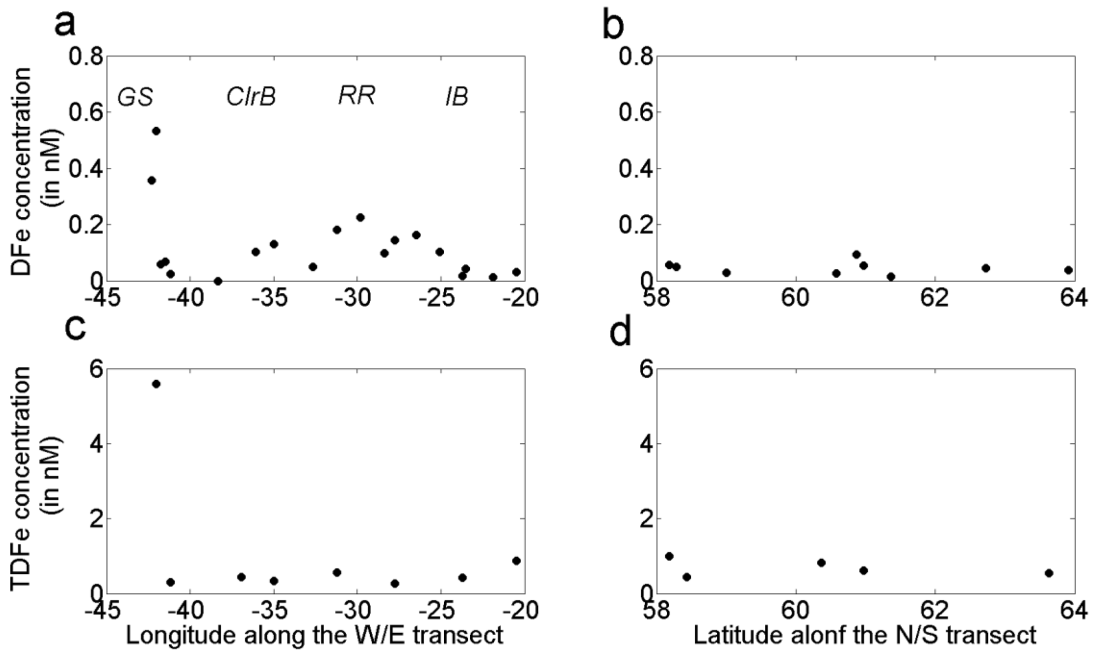

Figure S3: Surface underway (a, b) DFe and (c, d) TDFe concentration (in nM) along the 60ºN transect and along the -35ºW transect (see Figure S1) during D354. GS: Greenland Shelf; CIRB: Central Irminger Basin; RR: Reykjanes Ridge, IB: Iceland Basin.

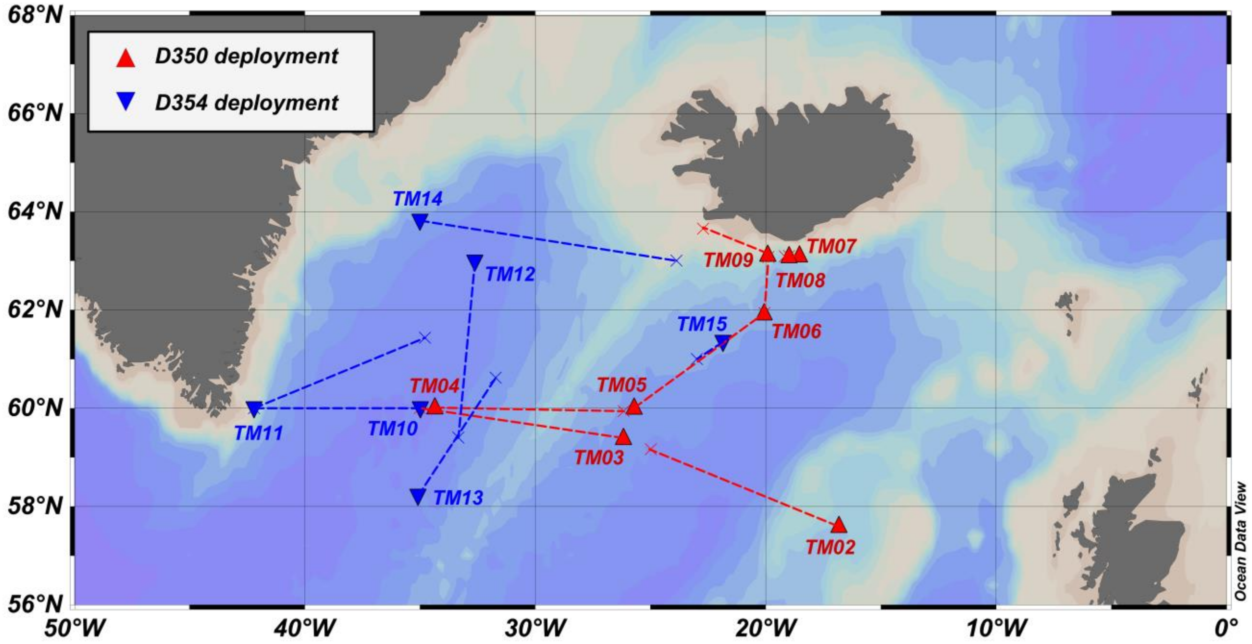

Figure S4: Aerosol sampling for D350 (red dotted line; spring cruise) and D354 (blue dotted line; summer cruise). Tiangles indicate position where aerosol sampling commenced, and crosses where sampling finished. Figure produced using Ocean Data View (Schlitzer, R., Ocean Data View, odv.awi.de, 2017).

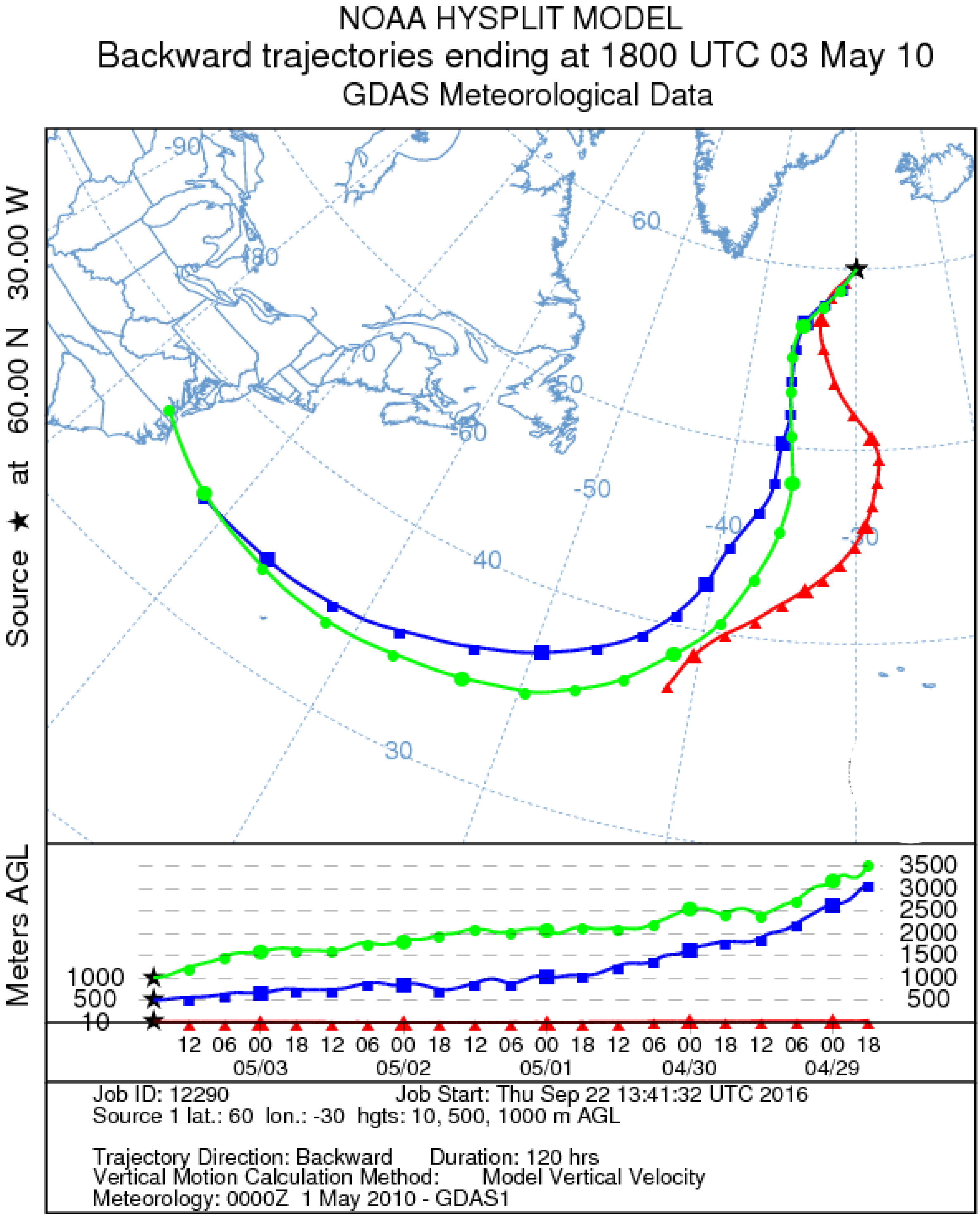

Figure S5: Airmass backtrajectories for 10, 500 and 1000 m height for a 5 day period ending May 3, 2010 (1800 h), related to the position of RRS *Discovery*. We acknowledge the US National Oceanic and Atmospheric Administration/Department of Commerce for the use of the Hysplit model and image.

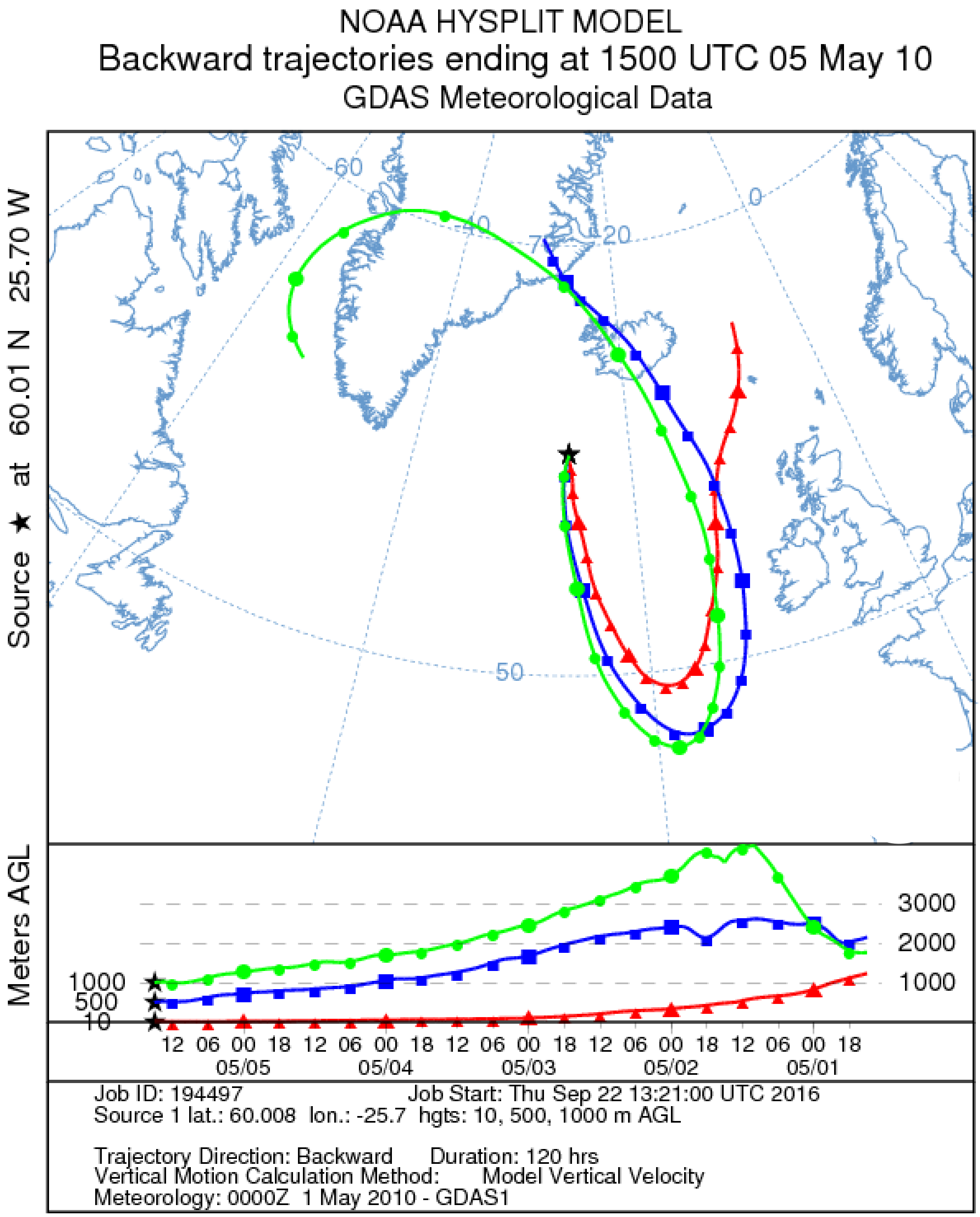

Figure S6: Airmass backtrajectories for 10, 500 and 1000 m height for a 5 day period ending May 5, 2010 (1500 h), related to the position of RRS *Discovery*. We acknowledge the US National Oceanic and Atmospheric Administration/Department of Commerce for the use of the Hysplit model and image.

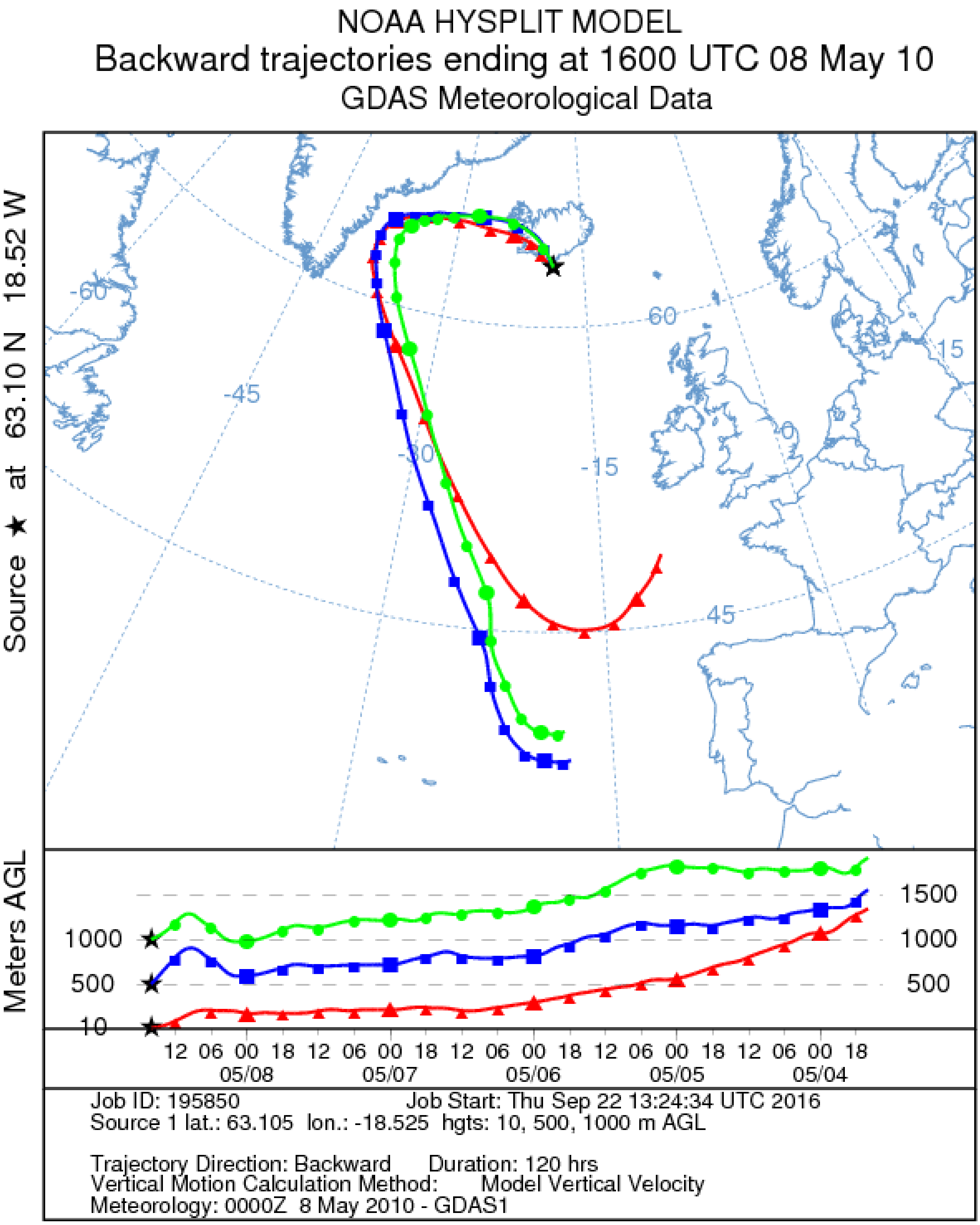

Figure S7: Airmass backtrajectories for 10, 500 and 1000 m height for a 5 day period ending May 8 2010 (1600 h), related to the position of RRS *Discovery*. We acknowledge the US National Oceanic and Atmospheric Administration/Department of Commerce for the use of the Hysplit model and image.

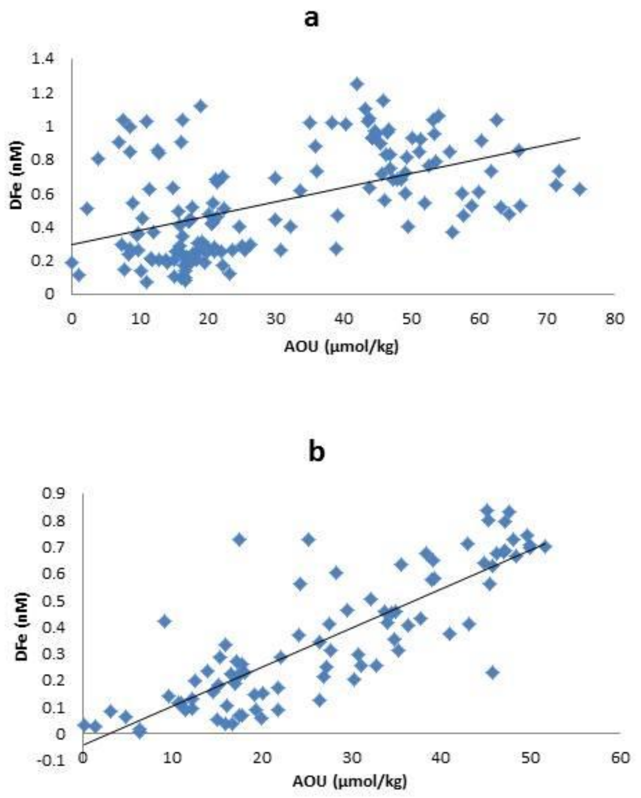

Figure S8: a. For stations in the IB, the DFe:AOU relationship with a slope of 0.0087±0.0012 nmol µmol^{-1} and an intercept of 0.29 ±0.04 nM (R^2 = 0.26, n=146). b. Stations in the IRB with a slope of 0.0150±0.0010 nmol µmol^{-1} and an intercept -0.03±0.033 nM (R^2 = 0.68, n=88).
